# SMYD5 regulates H4K20me3-marked heterochromatin to safeguard ES cell self-renewal and prevent spurious differentiation

**DOI:** 10.1186/s13072-017-0115-7

**Published:** 2017-02-23

**Authors:** Benjamin L. Kidder, Gangqing Hu, Kairong Cui, Keji Zhao

**Affiliations:** 1 Department of Oncology, Wayne State University School of Medicine, Detroit, MI USA; 2 Karmanos Cancer Institute, Wayne State University School of Medicine, Detroit, MI USA; 3 Systems Biology Center, National Heart, Lung and Blood Institute, National Institutes of Health, Bethesda, MD USA

**Keywords:** Embryonic stem cells, SMYD5, H4K20me3, Repetitive DNA, LTR, LINE, Pluripotent, Epigenetics, Chromatin, Heterochromatin, Genomics, RNA-Seq, ChIP-Seq, Self-renewal, Gene expression, Embryoid body, Differentiation, Histone methyltransferase

## Abstract

**Background:**

Epigenetic regulation of chromatin states is thought to control the self-renewal and differentiation of embryonic stem (ES) cells. However, the roles of repressive histone modifications such as trimethylated histone 4 lysine 20 (H4K20me3) in pluripotency and development are largely unknown.

**Results:**

Here, we show that the histone lysine methyltransferase SMYD5 mediates H4K20me3 at heterochromatin regions. Depletion of SMYD5 leads to compromised self-renewal, including dysregulated expression of OCT4 targets, and perturbed differentiation. SMYD5-bound regions are enriched with repetitive DNA elements. Knockdown of SMYD5 results in a global decrease of H4K20me3 levels, a redistribution of heterochromatin constituents including H3K9me3/2, G9a, and HP1α, and de-repression of endogenous retroelements. A loss of SMYD5-dependent silencing of heterochromatin nearby genic regions leads to upregulated expression of lineage-specific genes, thus contributing to the decreased self-renewal and perturbed differentiation of SMYD5-depleted ES cells.

**Conclusions:**

Altogether, these findings implicate a role for SMYD5 in regulating ES cell self-renewal and H4K20me3-marked heterochromatin.

**Electronic supplementary material:**

The online version of this article (doi:10.1186/s13072-017-0115-7) contains supplementary material, which is available to authorized users.

## Background

Compared to the extensive studies on active histone modifications, limited investigations have been performed on heterochromatic markers. Heterochromatic domains are generally inaccessible to DNA binding factors and transcriptionally silent [1]. Large regions of heterochromatin can be found around chromosomal structures such as centromeres and telomeres, while smaller domains are interspersed throughout the genome [2]. Heterochromatin plays a critical role in gene expression during development and differentiation [3] and is also involved in maintaining genome integrity by stabilizing repetitive DNA sequences throughout the genome by inhibiting recombination between homologous DNA repeats [4]. Heterochromatin is associated with H3K9 and H4K20 methylation. ESET/Setdb1 and LSD1, which control the methylation status of H3 lysine 9, are important for silencing of endogenous retroviruses (ERVs) in ES cells and during early embryogenesis [5, 6], suggesting critical roles of H3K9 methylation in ES cells. However, it remains unclear how H4K20me3 is regulated and how it contributes to the repression of endogenous retroelements in ES cells.

H4K20 methylation marks, which are evolutionarily conserved from yeast (*S. pombe*) to humans [7], have been implicated in having diverse cellular functions including the formation of heterochromatin, gene regulation and repression of transcription [8], DNA damage repair [9, 10], DNA replication [11], chromosome condensation [12], and genome stability [10, 13]. Although H4K20me1 is found in active genes [14, 15], H4K20me3 is associated with the formation of pericentric hetereochromatin by sequential methylation of H4K20me1 and H4K20me2 by Suv420h1 or Suv420h2, respectively [10, 16]. H4K20me3 marks repress transcription of repetitive elements [10, 17, 18]. SMYD5 has recently been shown to be a histone methyltransferase that mediates H4K20me3 modification in Drosophila and mouse primary macrophage cells [19]. However, the role of SMYD5 in mouse development, ES cell self-renewal and differentiation, and regulation of heterochromatin has not been fully elucidated.

In this study, we show that the H4K20me3 methyltransferase, SMYD5, targets H4K20me3 in heterochromatin regions containing retroelements and facilitates HP1α binding. Our results suggest that SMYD5 represses lineage-specific genes and thus contributes to the maintenance of ES cell lineage.

## Results

### SMYD5 regulates ES cell self-renewal

**Fig. 1:**
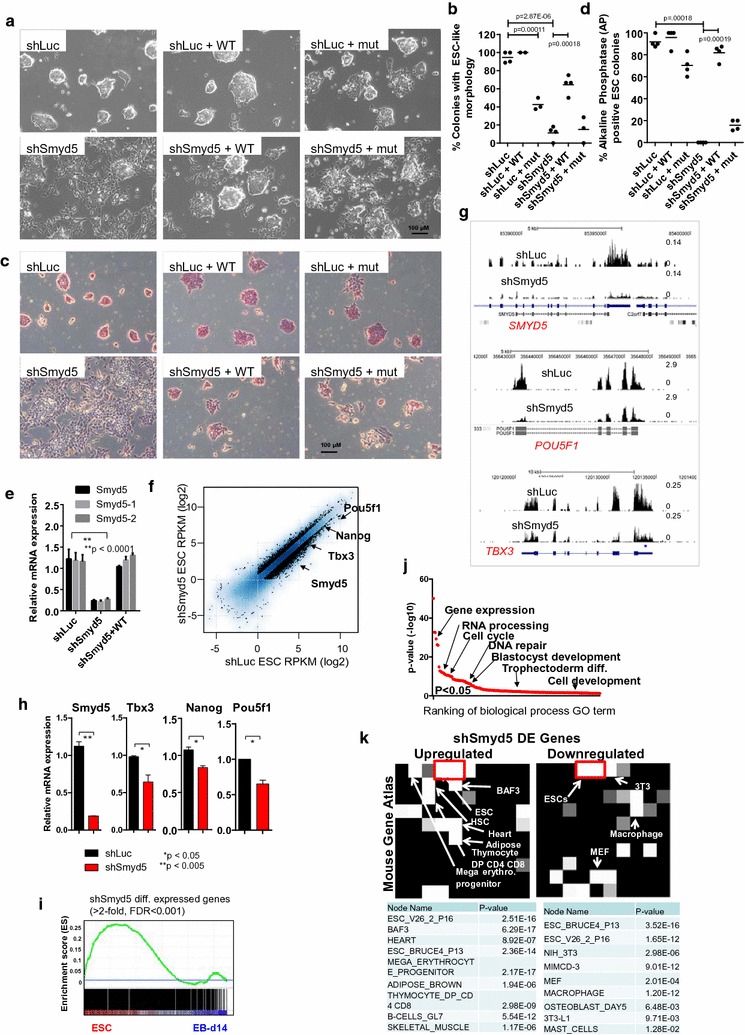
SMYD5 regulates ES cell self-renewal. **a** Bright-field microscopy of ES cells infected with shLuc or shSmyd5 lentiviral particles and wild-type (WT) SMYD5 or an enzymatically mutant (mut) version of SMYD5 (H315L and C317A) lentiviral particles and stably selected with puromycin and G418. **b** ES cell colonies were scored by morphology. The percentage of colonies with an ES-like morphology (compact and round vs. flattened) are represented as mean ± SEM. *P* values were calculated using a *t* test. **c** Alkaline phosphatase (AP) staining of ES cells. **d** ES cells were scored by AP staining. The percentage of AP positive colonies is represented as mean ± SEM. *p* values were calculated using a *t* test. **e** Quantitative RT-PCR (Q-RT-PCR) expression of SMYD5 using primers for three different regions of the SMYD5 coding region. **f** Scatter plot of RNA sequencing (RNA-Seq) gene expression analysis between shLuc and shSmyd5 ES cells. Log2 adjusted differentially expressed genes are plotted. Genes whose expression is greater than twofold (shLuc vs shSmyd5) and with an RPKM > 1 (reads per kilo bases of exon model per million reads) and FDR < 0.001 are shown in *black*. **g** UCSC genome browser view of differential expression of self-renewal genes in shSmyd5 and shLuc control ES cells. **h** Q-RT-PCR analysis of expression of Smyd5 and self-renewal genes in shLuc and shSmyd5 ES cells. **i** Gene set enrichment analysis (GSEA) [22] of differentially expressed genes in Smyd5 knockdown ES cells relative to undifferentiated and differentiated embryoid bodies (EBs). **j** Gene ontology (GO) functional annotation of differentially expressed genes analyzed using DAVID [23]. **k** Mouse gene atlas expression analysis evaluated using Network2Canvas [66] demonstrates that lineage and ES cell genes are misexpressed in shSmyd5 ES cells. Each node (*square*) represents a gene list (shLuc vs shSmyd5 DE genes) associated with a gene-set library (mouse gene atlas). The brightness (*white*) of each node is determined by its *p* value

Using RNA-Seq assays, we found that SMYD5 is highly expressed in ES cells and downregulated upon differentiation (see Additional file 1: Figure S1A) [20]. To study the role of SMYD5 in ES cell function, we knocked down *Smyd5* with lentiviral particles encoding three different short hairpin RNAs (shRNAs) (see Additional file 1: Figure S1B). Depletion of SMYD5 resulted in a loss of normal ES cell colony morphology (see Additional file 1: Figure S1C**)**, where shSmyd5 ES cell colonies became flattened and lost their tight cell–cell contact and became scattered at the colony periphery. The severity of phenotypes correlated with the knockdown efficiency (see Additional file 1: Figure S1C). To confirm the specificity of the shRNA sequences, we performed a rescue experiment by overexpressing an shRNA-resistant version of wild-type (WT) SMYD5 or an shRNA-resistant enzymatically mutant version of SMYD5 (H315L and C317A) [19]. Our results show that control ES cells (short hairpin luciferase—shLuc and shLuc + WT) maintained their colony morphology and overexpression of wild-type SMYD5 in short hairpin Smyd5 (shSmyd5) ES cells (shSmyd5 + WT) restored the 3D colony morphology of the majority of colonies to an ESC-like phenotype (Fig. 1a, b). While 99% of shLuc ES cell colonies exhibited an ESC-like morphology, only 11% of shSmyd5 ES cells remained intact (Fig. 1a, b). However, 70% of shSmyd5 ES cells overexpressing wild-type SMYD5 displayed an ESC-like morphology (Fig. 1b). In addition, while overexpression of mutant SMYD5 decreased the number of intact ESC-like colonies in shLuc ES cells (shLuc + mut) (Fig. 1a, b), the number of intact shSmyd5 ES cells (shSmyd5 + mut) did not significantly change, demonstrating that SMYD5 is important for ES cell self-renewal. Moreover, alkaline phosphatase (AP) staining, a marker of undifferentiated ES cells, was mostly absent in shSmyd5 ES cells or shSmyd5 ES cell colonies overexpressing mutant SMYD5 relative to control (shLuc) ES cells (Fig. 1c, d). However, AP staining was restored in 80% of shSmyd5 ES cells overexpressing wild-type SMYD5, further demonstrating that SMYD5 is important for ES cell self-renewal. In addition, we observed wild-type levels of SMYD5 expression in shSmyd5 ES cells overexpressing wild-type SMYD5 (Fig. 1e).

By comparing the global gene expression profiles of shSmyd5 and shLuc ES cells using RNA-Seq, we found 1616 genes differentially expressed (DE) at least twofold, and 4235 genes differentially expressed (DE) at least 1.5 fold, including the pluripotency regulators Oct4/*Pou5f1*, *Nanog*, and *Tbx3* (Fig. 1f), as exemplified by the UCSC genome browser tracks (Fig. 1g). Genes differentially expressed at least 1.5 fold were used for downstream analyses. Moreover, several stem cell genes, including *Esrrb* and *Tbx3*, were downregulated in *Smyd5* knockdown ES cells. Because ESRRB and TBX3 occupy promoter regions of other stem cell genes, including *Oct4* and *Nanog*, a reduction in their expression may further influence the self-renewal state of *Smyd5* knockdown ES cells. We confirmed the expression of several self-renewal genes using Q-RT-PCR (Fig. 1h). We then compared these DE genes with global expression data from ES cells and embryoid body (EB) differentiated cells [20], which emulates early embryo development [21] (see “Methods”), using gene set enrichment analysis (GSEA) [22]. This analysis shows that differentially expressed genes are enriched in ES cells (Fig. 1i), suggesting that a loss of SMYD5 impacts ES cell function. Moreover, DAVID [23] gene ontology (GO) terms enriched in DE genes include gene expression, cell cycle, RNA processing, DNA repair, blastocyst development, trophectoderm differentiation, and cell development (Fig. 1j). We also found that expression of many of the DE genes between shLuc and shSmyd5 ES cells is not only enriched in ES cells (Fig. 1k), but expression of many upregulated genes in shSmyd5 ES cells is also enriched in committed lineages, suggesting that expression of both lineage genes and ES cell regulators is impacted by depleting SMYD5. These results implicate a role for SMYD5 in repressing expression of lineage-specific genes in ES cells.

**Fig. 2:**
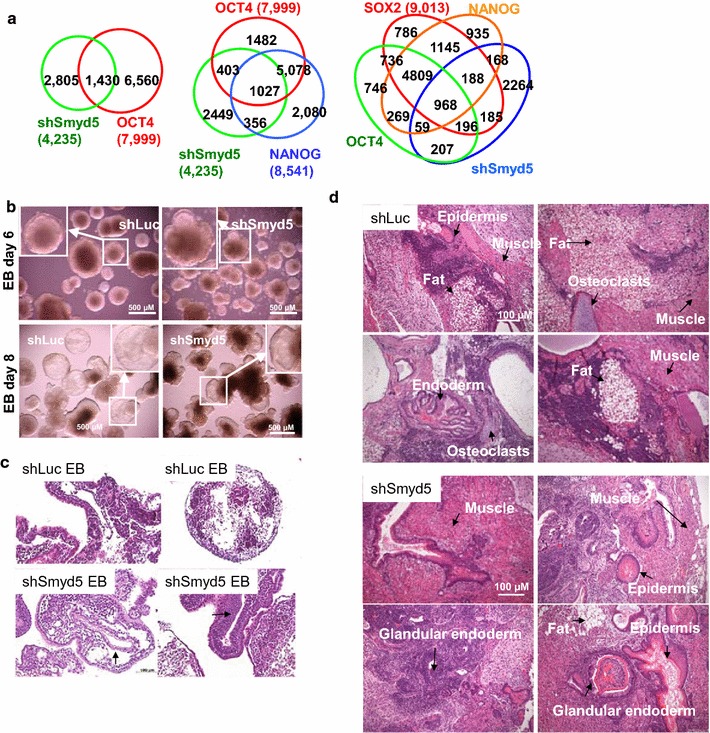
Altered differentiation of SMYD5-depleted ES cells. **a** Venn diagrams showing overlap between differentially expressed genes in shSmyd5 and shLuc ES cells and genes bound by OCT4, OCT4, and NANOG, or OCT4, SOX2, and NANOG. **b** Embryoid body (EB) formation shows abnormal differentiation of shSmyd5 ES cells. **c** Hematoxylin and eosin (H&E) histological sections of shLuc and shSmyd5 day 9 EBs. The *arrowheads* depict altered and advanced differentiation of shSmyd5 EBs relative to control (shLuc) EBs. The *bottom left panel* shows an EB with an atypical internal epithelial-like structure; the *bottom right panel* shows a thick epithelial-like layer. **d** H&E histological sections of teratomas generated from shLuc and shSmyd5 ES cells injected into SCID–beige mice. Tumors were harvested 4–6 weeks post-injection and evaluated using standard H&E histological methods. Transmitted white-light microscopy of sectioned teratomas. Heterogeneous differentiation of shLuc and shSmyd5 ES cells into ectoderm (keratinized epidermal cells), mesoderm (muscle and mesenchymal cells, adipocytes), and endoderm (glandular structures)

Because *Pou5f1* and *Nanog* are downregulated in shSmyd5 ES cells, we compared genes bound by OCT4, SOX2, and NANOG in ES cells [24] and genes misexpressed in shSmyd5 ES cells. We found that 34% of the DE genes are bound by OCT4 in ES cells (Fig. 2a), and OCT4, SOX2, and NANOG also co-occupied a number of SMYD5-regulated genes (Fig. 2a), suggesting that depletion of SMYD5 leads to perturbation of the core ES cell transcriptional circuitry.

### Altered differentiation of SMYD5-depleted ES cells

To investigate the function of SMYD5 during ES cell differentiation, we induced EB formation of shLuc and shSmyd5 ES cells in the absence of leukemia inhibitory factor (LIF). EB formation, which involves a change in culture condition from 2D to 3D, is a suitable assay for evaluating ES cell differentiation because it recapitulates embryogenesis [21, 25]. shLuc and shSmyd5 ES cells were cultured in the absence of LIF on low-attachment dishes to induce EB differentiation over two weeks. While shLuc ES cells formed mainly circular or globular EB structures containing a primitive endoderm (PE) layer during early differentiation (day 6) (Fig. 2b, top), shSmyd5 ES cells formed complex structures containing bulges lined with a PE layer (Fig. 2b, top). The PE layer forms in vivo by differentiation of cells located on the surface of the ICM facing the blastocoel [25]. This pattern was further pronounced at day 8 of EB differentiation (Fig. 2b, bottom). We also utilized teratoma formation assays to evaluate the in vivo differentiation ability of SMYD5-depleted ES cells into cells of the three germ layers (ectoderm, mesoderm, and endoderm). Hematoxylin and eosin (H&E) staining confirmed the presence of complex structures and more advanced differentiation in shSmyd5 EBs (Fig. 2c). A further evaluation of differentiation using teratoma formation revealed that while shSmyd5 and shLuc teratomas are both able to give rise to cells of the three germ layers including ectoderm (keratinized epithelium, epidermis), mesoderm (mesenchymal cells, muscle, adipocytes), and endoderm (glandular epithelium) (Fig. 2d), shSmyd5 teratomas had a greater presence of glandular endodermal cells.

### Knockdown of SMYD5 leads to accelerated gene expression changes during ES cell differentiation

**Fig. 3:**
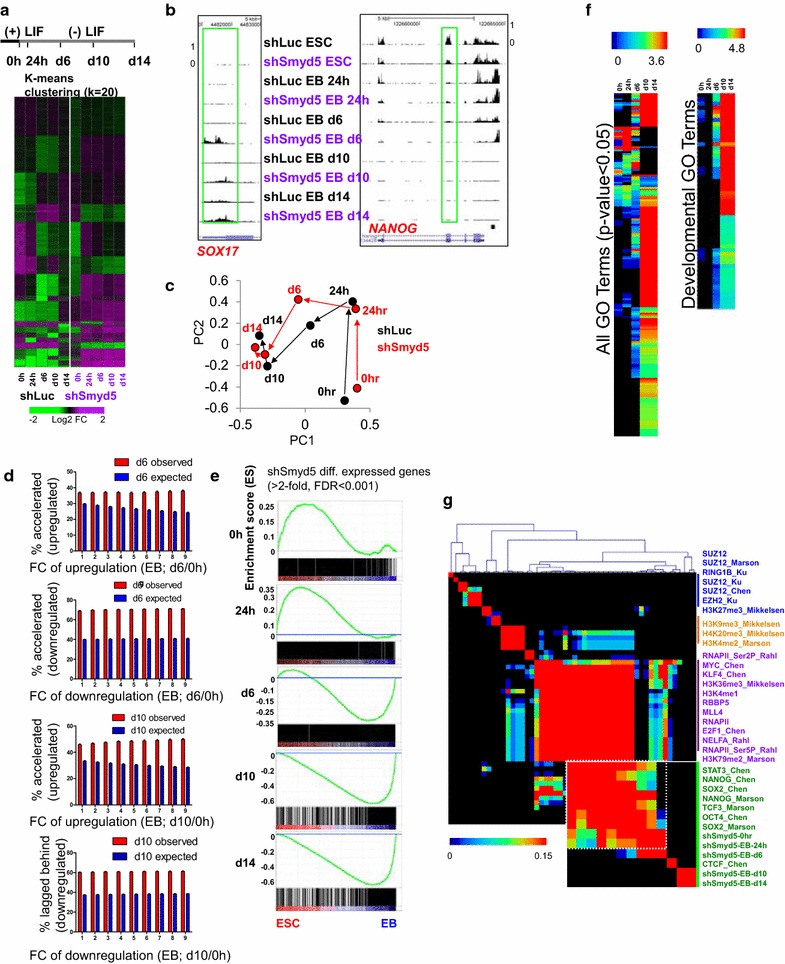
Transcriptome analysis reveals altered differentiation of SMYD5-depleted ES cells. **a** K-means clustering analysis of RNA-Seq data from shLuc and shSmyd5 ES cells differentiated without LIF for 14 days. The experimental design is shown on *top*. Differentially expressed genes (>twofold; RPKM > 1) clustered according to k-means. **b** Custom tracks of RNA-Seq data in the UCSC genome browser for undifferentiated and differentiated shLuc control and shSmyd5 ES cells. **c** Principal component analysis (PCA) of differentially expressed genes during EB differentiation of shSmyd5 and shLuc ES cells. **d** Prediction of differentially expressed genes due to chance or altered differentiation. The percentage of genes that lag behind during differentiation of shSmyd5 ES cells is less than expected. *Top each bar* represents a group of genes upregulated by at least alpha-fold (*X* axis) from ESC (0 h) to EB day 6 in the control cells. The percentage of genes with expression values that follow the order: EB day 6 (shLuc) > EB day 6 (shSmyd5) > ES cell is calculated (observed; *red bars*); *error bars* are generated by bootstrapping. The expression values of all genes are randomly shuffled independently for EB day 6 (shLuc), EB day 6 (shSmyd5), and ES cells and are repeated many times to give the means and standard deviations for the expectations (expected; *blue bars*). The *red bars* represent observed data. *Bottom each bar* represents a group of genes upregulated by at least alpha-fold (*X* axis) from ESC (0 h) to EB day 10 in the control cells. The percentage of genes with expression values that follow the order: EB day 10 (shLuc) > EB day 10 (shSmyd5) > ES cell is calculated (observed; *red bars*); *error bars* are generated by bootstrapping. The expression values of all genes are randomly shuffled independently for EB day 10 (shLuc), EB day 10 (shSmyd5), and ES cells and are repeated many times to give the means and standard deviations for the expectations (expected; *blue bars*). The *red bars* represent observed data. **e** Gene set enrichment analysis (GSEA) of differentially expressed genes during differentiation of shSmyd5 ES cells relative to ES cells and day 14 EBs. **f** DAVID gene ontology analysis of differentially expressed genes between shLuc and shSmyd5 ES cells and during EB differentiation. The hierarchical clustering heat map on the right shows enrichment of developmental GO terms. **g** Correlation matrix of differentially expressed (DE) genes during shSmyd5 ES cell differentiation with promoter binding of transcription factors and epigenetic modifiers. Heat map generated by evaluating pair-wise affinities between differentially expressed (DE) genes during shLuc and shSmyd5 EB differentiation using RNA-Seq datasets generated from this study (0, 24 h, 6, 10, 14 days) and published ChIP-Seq data [14, 24, 27–29, 67]. AutoSOME [68] was used to generate pair-wise affinity values

To identify gene expression defects caused by depletion of SMYD5, we evaluated DE genes during differentiation of shLuc and shSmyd5 ES cells (Fig. 3a) using RNA-Seq. K-means clustering (*k* = 20) was used to identify patterns of gene expression variability (Fig. 3a). These results highlight clusters of genes such as self-renewal genes, including *Nanog* (Fig. 3a, b; see Additional file 1: Figure S1D), that were rapidly downregulated in shSmyd5 EBs, and lineage-specific genes, such as *Sox17*, *Afp*, *Gata6*, and *Tbx5* (Fig. 3a, b; see Additional file 1: Figure S1D), that were differentially expressed between shSmyd5 and shLuc EBs. Because Sox17 is a transcription factor that is expressed in endodermal lineages, a driver of extraembryonic endoderm transcriptional programs, and is important in antagonizing expression of *Pou5f1* and *Nanog* during differentiation [26], its upregulation during differentiation of SMYD5-depleted ES cells may explain the presence of complex structures involving primitive endoderm. To compare the transcriptomes of shSmyd5 EBs relative to shLuc EBs, we used principal component analysis (PCA), which showed that shSmyd5 EBs progress through the first two components at an altered trajectory (Fig. 3c), suggesting that knockdown of *Smyd5* leads to perturbed differentiation.

To further address the phenomenon of altered differentiation, we built a predictive model to determine the probability of expression changes due to chance or altered differentiation following differentiation of shSmyd5 EBs. Expression changes that indicate altered differentiation include genes that show altered downregulation or upregulation during EB differentiation. Our findings demonstrate that the percentage of upregulated or downregulated genes outpaced the expected (Fig. 3d, see “Methods”), demonstrating that shSmyd5 EBs exhibit altered differentiation. The *x-*axis represents genes that are upregulated or downregulated by alpha-fold from ES cells (d0 of differentiation) to day 6 EBs, or from ES cells to day 10 EBs.

GSEA was then used to investigate the expression state of DE genes during differentiation in the absence of SMYD5. Our results demonstrate that ES cell-enriched genes are differentially expressed during early differentiation (Fig. 3e, top graphs) while EB-enriched genes are differentially expressed later in differentiation (Fig. 3e, bottom graphs). DAVID GO analysis revealed that many developmental GO terms were overrepresented during later EB differentiation (Fig. 3f), suggesting that SMYD5 regulates developmental genes during differentiation.

Because depletion of SMYD5 resulted in the differential expression of many OCT4, SOX2, and NANOG targets (Fig. 2a), we evaluated whether DE genes during shSmyd5 EB differentiation were also occupied by ES cell-enriched transcription regulators or marked by histone modifications using public datasets [14, 24, 27–29] (Fig. 3g). In shSmyd5 ES cells and during early EB formation (24 h), we observed a strong correlation between DE genes and binding of ES cell-enriched factors OCT4, SOX2, NANOG, and STAT3 (Fig. 3g), suggesting that depletion of SMYD5 leads to the dysregulated expression of pluripotency-regulator targets during early EB differentiation.

### SMYD5 mediates H4K20me3 modification in ES cells

**Fig. 4:**
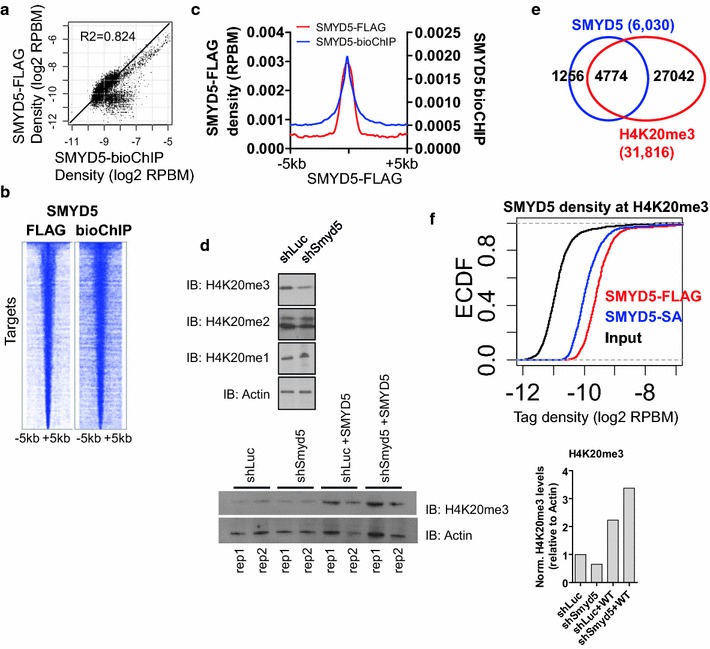
SMYD5 and trimethylated histone co-occupy genomic regions in ES cells. **a** Comparison of SMYD5-bioChIP and SMYD5-FLAG ChIP-Seq peaks. Scatter plot of log2 SMYD5 density at ChIP-enriched regions. **b** Heat map of SMYD5 ChIP-Seq densities. **c** Average profiles of SMYD5-bioChIP and SMYD5-FLAG density at SMYD5-FLAG enriched regions. **d** Western blot of H4K20me3, H4K20me2, and H4K20me1 in shLuc and shSmyd5 ES cells (*top*), and H4K20me3 in shLuc, shSmyd5, shLuc + WT, and shSmyd5 + WT (*bottom*). The bar graph (*bottom right*) shows H4K20me3 levels normalized to actin using ImageJ software (https://imagej.nih.gov/ij/). **e** Comparison of SMYD5 and H4K20me3 ChIP-Seq peaks. **f** Empirical cumulative distribution function (ECDF) for SMYD5-FLAG and SMYD5-bioChIP density at H4K20me3-enriched regions in ES cells

To evaluate the genome-wide distribution of SMYD5 in ES cells, we utilized biotin-mediated ChIP-Seq (bioChIP-Seq) [30, 31] and FLAG ChIP-Seq as described in the materials and methods. Using this approach, we observed a high overlap of SMYD5 binding using these two approaches (see Additional file 2: Figure S2A) by evaluating the density of SMYD5 at “Spatial Clustering for Identification of ChIP-Enriched Regions” (SICER) islands (see “Methods”) (Fig. 4a) as well as by heat maps (Fig. 4b; see Additional file 2: Figure S2B) and average profiles (Fig. 4c; see Additional file 2: Figure S2C).

Because SMYD5 has been shown to deposit H4K20me3 marks [19], we evaluated global levels of H4K20 methylation in *Smyd5* knockdown ES cells using western blotting. Our results show that depletion of SMYD5 led to decreased H4K20me3, but not H4K20me2 or H4K20me1 (Fig. 4d; see Additional file 2: Figure S2D), demonstrating that SMYD5 confers H4K20me3 methyltransferase activity. We also observed a restoration of H4K20me3 levels in shSmyd5 ES cells overexpressing an shRNA-resistant version of SMYD5 (Fig. 4d, bottom right). Moreover, our ChIP-Seq data indicated that a majority of SMYD5 occupied regions (79%) contain H4K20me3 marks (Fig. 4e), where SMYD5 binding is significantly enriched in H4K20me3 islands (Fig. 4f). Overall, these results demonstrate that SMYD5 occupies chromatin regions containing H4K20me3.

### SMYD5 and H4K20me3 co-occupy repetitive DNA elements

Since H4K20me3 is known to be enriched in repetitive sequences [14], we analyzed the enrichment of DNA repeats in SMYD5 islands. To this end, we evaluated the percent coverage of H4K20me3 or SMYD5 peaks that overlap a repeat element. We observed enrichment of long interspersed elements (LINE) and long-terminal repeat (LTR) elements in H4K20me3 (see Additional file 3: Figure S3A, top) and SMYD5 regions (see Additional file 3: Figure S3A, bottom), respectively. Similarly, LINE and LTR elements were enriched in H3K9me3 regions (see Additional file 3: Figure S3A, middle panel), consistent with the co-localization of H3K9me3 and H4K20me3 on chromatin. Enrichment of LINE and LTR elements was markedly higher at these regions relative to random genomic sequences of comparable size and frequency, and H3K4me3 regions, which were used as controls, demonstrating that SMYD5 and H4K20me3 islands are enriched at LINE and LTR repetitive DNA elements.

**Fig. 5:**
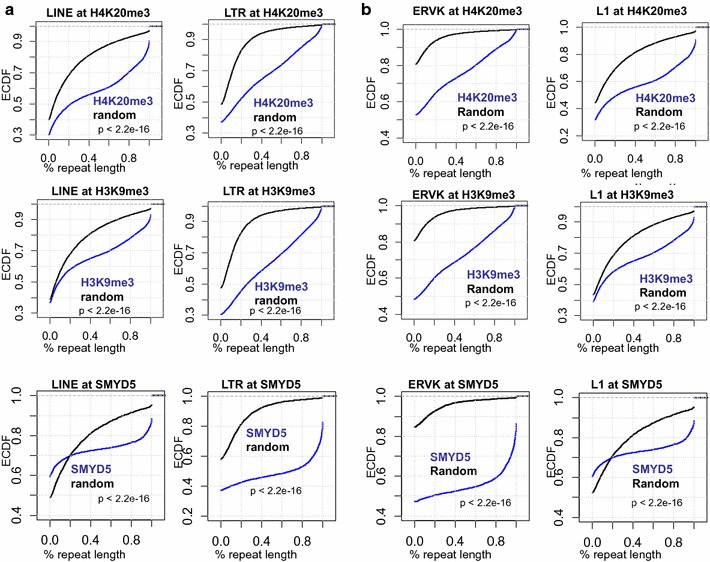
SMYD5 and H4K20me3 occupy repetitive DNA elements in ES cells. Comparison of H4K20me3, H3K9me3, and SMYD5 enriched sequences and annotated repetitive sequences (http://www.repeatmasker.org). Empirical cumulative distribution (ECDF) for the percent coverage of a **a** LINE or LTR repeat class or **b** family member (L1 or ERVK) across all H4K20me3 (*top*) or H3K9me3 (*middle*) islands, or SMYD5 regions (*bottom*) relative to random genomic regions (*black*). *Y-*axis shows the percentage of genes that exhibit a percent repeat length less than the value specified by the *x-*axis. A *line* shifted to the right means a systematic increase in the percent coverage of a repeat element in ChIP-Seq peaks relative to random genomic sequences. *p* value for all <2.2e−16 (Kolmogorov–Smirnov test)

We also evaluated the percent coverage of LINE and LTR sequences for all SMYD5, H4K20me3, and H3K9me3 islands. Using this approach, we observed enrichment of LINE and LTR sequences in H4K20me3, SMYD5, and H3K9me3 regions relative to random genomic regions (Fig. 5a).

To investigate which repeat family members are enriched in H4K20me3, H3K9me3, or SMYD5 regions, we evaluated the percentage of peaks that overlap a repeat family element. We observed enrichment of ERVK (LTR class) and L1 (LINE class) family repetitive elements in H4K20me3 (see Additional file 3: Figure S3B, top), H3K9me3 (see Additional file 3: Figure S3B, middle), and SMYD5 regions **(**see Additional file 3: Figure S3B, bottom**)**, respectively. We also evaluated the percent coverage of ERVK and L1 sequences for all SMYD5, H4K20me3, and H3K9me3 islands. Using this approach, we observed enrichment of ERVK and L1 sequences in H4K20me3, H3K9me3, and SMYD5 regions relative to random genomic regions (Fig. 5b), To gain further insight into which specific repeats are enriched in SMYD5, H4K20me3, and H3K9me3 regions, we evaluated the percentage of peaks that overlap repeat subfamilies within the LINE or LTR repeat class. Of the hundreds of annotated repeat subfamilies that we surveyed, only a few repeat subfamilies were found to be enriched within these regions (see Additional file 3: Figure S3C). While several L1Md repeats (L1Md_T, L1Md_A) are enriched in H4K20me3, H3K9me3, and SMYD5 occupied regions, two repeats (L1Md_F and L1Md_F2) were only enriched in H4K20me3 and H3K9me3 marked regions (see Additional file 3: Figure S3C).

By clustering repeat subfamilies by their enrichment in SMYD5, H4K20me3, or H3K9me3 regions, our results further demonstrate that a few LINE/LTR repeat subfamilies are enriched within H4K20me3 (see Additional file 4: Figure S4A), H3K9me3 (see Additional file 4: Figure S4B), and SMYD5 regions (see Additional file 4: Figure S4C).

### Knockdown of SMYD5 leads to decreased levels of H4K20me3, H3K9me3/2, and HP1α binding

**Fig. 6:**
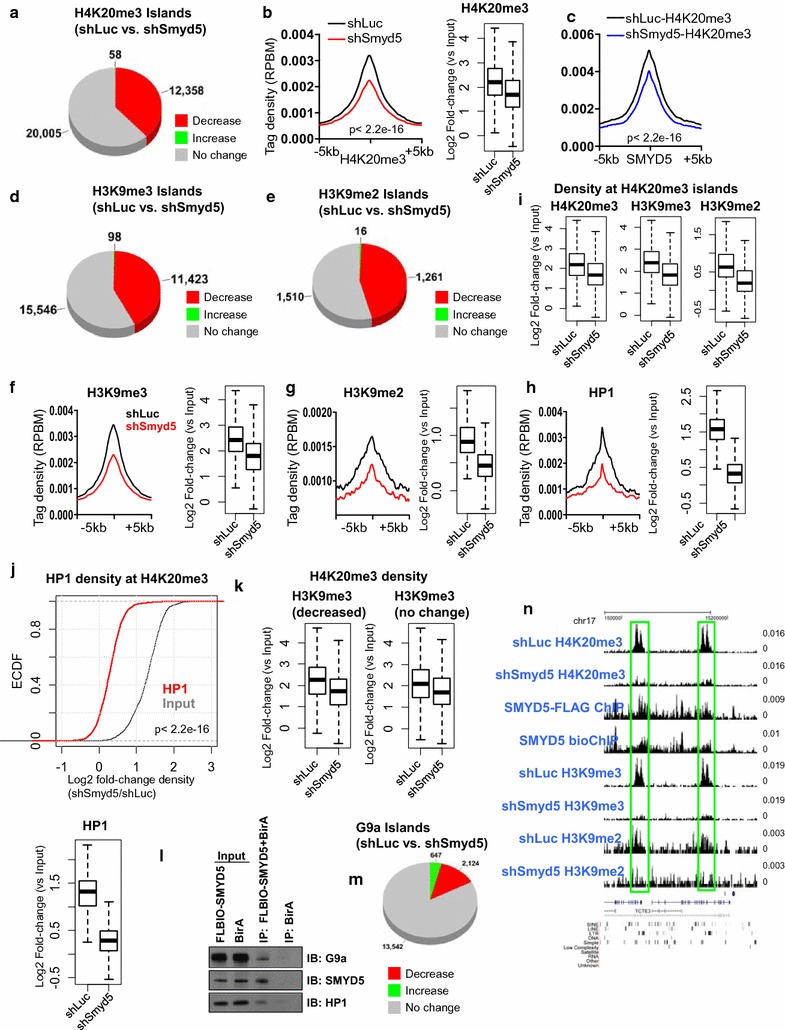
Depletion of SMYD5 leads to decreased H4K20me3, H3K9me3/2, and HP1α binding. **a** Change in the global distribution of H4K20me3 in shSmyd5 ES cells. **b** Average profile and *boxplot* of H4K20me3 ChIP-Seq tag density in shSmyd5 ES cells. **c** Average profiles of H4K20me3 at SMYD5-enriched regions in shLuc and shSmyd5 ES cells. **d**, **e** Changes in global distributions of **d** H3K9me3 (**e**) and H3K9me2 in shSmyd5 ES cells relative to shLuc ES cells. **f**–**h** Average profiles and *boxplot* of **f** H3K9me3, **g** H3K9me2, and **h** HP1α densities in shLuc and shSmyd5 ES cells. **i** Boxplot depicting density of H4K20me3, H3K9me3, and H3K9me2 at H4K20me3 islands. **j** Empirical cumulative distribution (ECDF) for the fold-change in density of HP1α in shSmyd5 ES cells. The *red line* shifted to the *left* of the input (*gray*) shows a systematic decrease in enrichment in shSmyd5 ES cells. *Boxplot* below shows HP1α density (log2 fold-change vs. input) in shLuc and shSmyd5 ES cells. **k** H4K20me3 density at regions at regions with decreased or unaltered H3K9me3 levels. **l** SMYD5 associates with HP1α and G9a. FLBIO-SMYD5 (biotinylated SMYD5 + BirA) or BirA (control) ES cells were used to immunoprecipitate SMYD5 protein with avidin-agarose beads. Immunoprecipitates were analyzed by immunoblotting with anti-HP1α, anti-G9a, and anti-SMYD5 antibodies. **m** Changes in the global distribution of G9a in shSmyd5 ES cells relative to shLuc ES cells. **n** Altered profiles of H4K20me3 at SMYD5-enriched regions (H2-Q1, H2-Q7) in shLuc and shSmyd5 ES cells

To further directly test whether SMYD5 regulates H4K20me3 in ES cells, we investigated the global distribution of H4K20me3 in shSmyd5 ES cells using ChIP-Seq. Our results revealed that 12,358 islands showed a decrease in H4K20me3 levels in shSmyd5 ES cells (FDR < 0.001, fold-change >1.5) (Fig. 6a). A comparison of average H4K20me3 profiles around H4K20me3 peaks (Fig. 6b, left panel), and a boxplot (Fig. 6b, right panel), also showed global decreases in H4K20me3 levels in shSmyd5 ES cells compared with shLuc ES cells (*p* < 2.2e−16). Similarly, the levels of H4K20me3 at SMYD5-enriched regions also decreased in shSmyd5 ES cells (*p* < 2.2e−16) (Fig. 6c; see Additional file 5: Figure S5), consistent with a role for SMYD5 in regulating H4K20me3 on chromatin.

To determine whether both H4K20me3 and SMYD5 are simultaneously present at the same genomic regions, we performed Re-ChIP, also termed sequential ChIP [32], by immunoprecipitating ES cell chromatin first with an H4K20me3 or a FLAG antibody (for SMYD5), and second with a FLAG or H4K20me3 antibody, respectively. We then performed reChIP-PCR and found that each was significantly enriched relative to the control, Nanog promoter, which is not enriched with SMYD5 or H4K20me3 (see Additional file 6: Figure S6A). For example, H4K20me3 + SMYD5 reChIP levels were enriched >10–25 fold (relative to control; Nanog promoter) at H4K20me3/SMYD5 co-occupied regions, and SMYD5 + H4K20me3 reChIP levels were also elevated ~6–11 fold (relative to the control) at H4K20me3/SMYD5 marked regions (see Additional file 6: Figure S6B-C), demonstrating that a subset of H4K20me3 marked regions contain SMYD5 marks.

To test whether SMYD5-mediated H4K20me3 affects H3K9 methylation at heterochromatin regions, we evaluated the global distributions of H3K9me3 and H3K9me2 in *Smyd5* knockdown cells. These results show that levels decreased at a subset of H3K9me3 (Fig. 6d) and H3K9me2 islands (Fig. 6e) in shSmyd5 ES cells compared with shLuc ES cells. A comparison of average profiles and boxplots of H3K9me3 (Fig. 6f) or H3K9me2 (Fig. 6g) revealed decreased H3K9me3/2 enrichment, suggesting that the H4K20me3 signal may be important for the H3K9me3 modification in heterochromatin in ES cells. Our results show that 72% of H3K9me3 islands overlap with H4K20me3 islands, and 70% of SMYD5 islands overlap with H3K9me3 islands. In addition, 48% of SMYD5 islands exhibit decreased H3K9me3 levels (>1.5 fold-change, FDR < 0.001), while 36% of all H3K9me3 islands showed decreased levels in SMYD5-depleted ES cells. These results suggest that SMYD5-bound regions are more likely to exhibit decreased H3K9me3 levels relative to regions without SMYD5 binding in SMYD5-depleted cells.

Because H3K9 methylation is important for the recruitment of the heterochromatin protein, HP1 [33], we examined HP1α binding profiles in shSmyd5 ES cells using average profiles (Fig. 6h, left panel) and a boxplot (Fig. 6h, right panel). Our data indicate that HP1α levels decrease in shSmyd5 ES cells, suggesting that depletion of SMYD5 leads to decreased heterochromatin. To test whether decreases in H3K9me3/2 and HP1α correlate with decreases in H4K20me3 levels, we compared their changes at H4K20me3 islands relative to input chromatin. These results show that H3K9me3/2 **(**Fig. 6i) and HP1α levels **(**Fig. 6j) decrease at H4K20me3 islands in shSmyd5 ES cells. We also surveyed H4K20me3 levels at H3K9me3 marked regions in SMYD5-depleted ES cells and observed greater decreases in H4K20me3 at regions where H3K9me3 decreased relative to regions where H3K9me3 levels did not change significantly (*p* < 2.2e−16) (Fig. 6k), suggesting that decreases in H4K20me3 are correlated with decreased H3K9me3 levels.

To investigate how SMYD5-mediated decreases in H4K20me3 affect H3K9me3 levels, we hypothesized that H3K9 methyltransferases may bind H4K20me3 marks. However, while we observed binding of G9a, HP1α, and ESET to H3K9me3/2 using an in vitro pull-down assay with biotinylated histone H4 and H3 peptides and nuclear extracts from ES cells, we did not observe binding to H4K20me3/2/1 (see Additional file 7: Figure S7A). To investigate whether SMYD5 directly interacts with heterochromatin constituents HP1α and G9a, we immunoprecipitated SMYD5 and performed immunoblotting using anti-HP1α, anti-G9a, and anti-SMYD5 antibodies. Our results show that SMYD5 binds to HP1α and G9a (Fig. 6l). Moreover, using ChIP-Seq analysis to survey global levels of G9a, we found that G9a levels decrease in shSmyd5 ES cells relative to shLuc ES cells (Fig. 6m). Our results also show that G9a occupies 40% of SMYD5 islands. Overall, these results suggest that SMYD5 interacts with heterochromatin proteins HP1α and G9a, and depletion of SMYD5 leads to decreased binding of HP1α and G9a. In addition, inspection of custom tracks on the UCSC genome browser revealed decreased levels of H4K20me3 and H3K9me3 at several SMYD5 occupied regions (Fig. 6n).

To investigate whether enrichment of repressive histone marks and associated chromatin factors decreases at LINE and LTR regions in the absence of SMYD5, we compared the densities of H4K20me3, H3K9me3, and HP1α at LINE (see Additional file 7: Figure S7B) and LTR (see Additional file 7: Figure S7C) regions. These results demonstrate that H4K20me3, H3K9me3, and HP1α levels decrease at LINE (see Additional file 7: Figure S7B) and LTR (see Additional file 7: Figure S7C) regions in shSmyd5 ES cells. We also observed enrichment of SMYD5 at LINE (see Additional file 7: Figure S7B) and LTR (see Additional file 7: Figure S7C) regions relative to Input DNA in ES cells. Overall, these results suggest that SMYD5 occupies LINE and LTR repetitive DNA elements and catalyzes H4K20me3 modifications, which are important for H3K9me3 modifications and HP1α binding in the heterochromatic regions.

### Increased expression of repetitive DNA in SMYD5 depleted ES cells

**Fig. 7:**
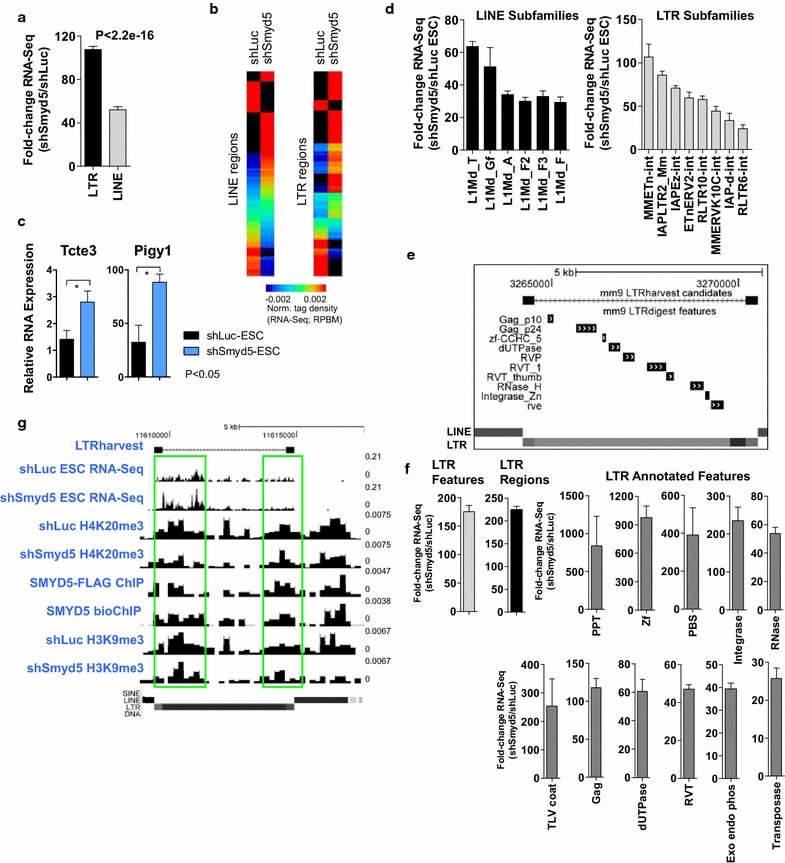
Elevated expression of repetitive DNA elements in shSmyd5 ES cells. **a** Fold-change expression of LINE/LTR repetitive DNA sequences in shSmyd5 ES cells relative to shLuc ES cells. *p* value for all <2.2e−16 (Kolmogorov–Smirnov test). **b** Heat map showing expression of a subset of LINE and LTR regions in shLuc and shSmyd5 ES cells. **c** Q-RT-PCR expression analysis of two LINE elements (*p* value <0.05). **d** Fold-change expression of LINE (*left*) and LTR (*right*) repeat subfamilies in shLuc and shSmyd5 ES cells. **e** De novo search for LTR retrotransposons/ERVs in the mouse genome (mm9) using LTRharvest software, and annotated using LTRdigest software. A representative full-length region with internal features is shown. **f** Fold-change expression of LTR internal features and LTR UTR regions between shLuc and shSmyd5 ES cells. **g** Browser view of RNA-Seq expression and H4K20me3, and H3K9me3 in shLuc and shSmyd5 ES cells, and SMYD5-FLAG and SMYD5-bioChIP in ES cells

To investigate whether SMYD5-mediated H4K20me3 plays a role in silencing of LINE, and LTR elements, we evaluated their expression in shLuc and shSmyd5 ES cells (Fig. 7a). These results revealed a marked increase in expression of LINE and LTR elements in shSmyd5 ES cells (Fig. 7a; *p* < 2.2e−16). We then applied a stringent filter to identify LINE and LTR regions (>0.003 RPBM tag density per site) and evaluated the RNA-Seq expression profile of this subset of LINE and LTR regions. Heat maps revealed an overall increase in expression of both LINE and LTR elements (Fig. 7b), suggesting that a loss of SMYD5 leads to global increases in expression of the LINE and LTR repetitive sequences. We confirmed the increased expression of several LINE regions, using Q-RT-PCR (Fig. 7c) and RNA-Seq. We also investigated the expression of LINE and LTR repeats in shLuc and shSmyd5 ES cells and observed de-repression of LTR repeat subfamilies, which are enriched in H4K20me3- and SMYD5-bound regions, including MMETn, IAPLTR2_Mm, IAPEz-int, ETnERV2-int, RLTR10-int, MMERK10C-int, IAP-d-int, RLTR6-int (MMETn, 107-fold increase; IAPLTR2_Mm, 86-fold increase; IAPEz-int, 71-fold increase), and de-repression of LINE repeat subfamilies, including L1Md_T, L1Md_Gf, L1Md_A, L1Md_F2, L1Md_F3, and L1Md_F in SMYD5-depleted ES cells (L1Md_T, 79-fold increase; L1Md_Gf, 51-fold increase; L1Md_T, 79-fold increase) (Fig. 7d).

Because we observed de-repression of LTR and LINE subfamilies in *Smyd5* knockdown ES cells, we reasoned that full-length, or intact, LTR retrotransposons and ERVs may be de-repressed in SMYD5-depleted ES cells. To test this possibility, we performed a de novo search for full-length LTR retrotransposons and ERVs in the mouse genome using LTRharvest software [34], which provides annotations of known LTR features. Using this approach, we identified 11,394 full-length LTR retrotransposons/ERVs in the mouse genome. We then annotated these LTR regions using LTRdigest software [35] and identified 20,852 internal features including sequences encoding viral proteins such as gag and pol (Fig. 7e). An evaluation of the expression state of these LTR features revealed an overall increase in the expression of full-length (intact) LTR/ERV annotated features in SMYD5-depleted ES cells (Fig. 7f).

To investigate a relationship between the de-repression of LTRs/ERV regions and occupancy of SMYD5/H4K20me3, we evaluated the overlap between LTR regions and SMYD5/H4K20me3 occupancy. Using this method, we found 872 regions which were occupied by SMYD5/H4K20me3 and contained LTR/ERV sequences. An examination of custom UCSC genome browser tracks revealed SMYD5 binding at a representative region containing LTR and LINE elements accompanied by decreased levels of H4K20me3, H3K9me3, and H3K9me2 in shSmyd5 ES cells relative to shLuc ES cells (Fig. 7g; see Additional file 8: Figure S8).

To investigate whether loss of SMYD5-dependent silencing of LTR/ERV elements leads to upregulated expression of nearby genes, which was observed in ESET/Setdb1 knockout ES cells [36], we first evaluated the number of upregulated genes in SMYD5 knockdown ES cells that contain LTR/ERV sequences within 10 kb of their TSS (see Additional file 9: Figure S9A-B). Annotation of these LTR/ERV elements revealed that they mainly reside in intronic and intergenic regions (see Additional file 9: Figure S9C). We then evaluated the expression state of LTR/ERV elements nearby differentially expressed genes. These results revealed an increase in expression of LTR/ERV elements in SMYD5 knockdown ES cells relative to control ES cells (see Additional file 9: Figure S9D). Moreover, we also observed decreased H4K20me3 levels at nearby islands in SMYD5 knockdown ES cells (see Additional file 9: Figure S9E), suggesting that SMYD5-dependent control of H4K20me3 supports the repression of LTR/ERV elements of nearby genes. We then investigated whether upregulated genes in shSmyd5 ES cells contain SMYD5 binding and LTR/LINE elements within 10 kb of their TSS are lineage-specific. Indeed, our results show that expression of lineage-specific genes bound by SMYD5 and containing LTR/LINE elements is upregulated in shSmyd5 ES cells (see Additional file 9: Figure S9F). These results suggest that SMYD5-dependent silencing of LTR/LINE elements represses expression of lineage-specific genes in ES cells. Overall, these results demonstrate that SMYD5 influences gene expression of nearby genes by silencing LTR/ERV elements.

## Discussion

### SMYD5 is important for ES cell self-renewal and differentiation

ES cell self-renewal is governed by networks of transcription factors, including OCT4, SOX2, NANOG, and TBX3 [27, 37, 38], and epigenetic regulators such as BRG1 [39, 40] and KDM5B [20, 41] that participate in regulating transcription of genes that promote self-renewal while repressing developmental genes. Disruption of these factors abrogates self-renewal leading to specific or mixed-lineage differentiation. While many studies have focused on the roles of chromatin modifying enzymes that regulate active marks such as H3K4 methylation [20, 41–43], or repressive histone marks such as H3K27 or H3K9 methylation [44–48], few regulators of the repressive histone mark H4K20me3 have been shown to be important for mouse development [10, 13], and none have been shown to be important for ES cell self-renewal. In this study, we have provided evidence that SMYD5, which mediates H4K20me3 marks, is a critical regulator of ES cell function. We found that knockdown of *Smyd5* resulted in decreased ES cell colony integrity and decreased expression of pluripotency regulators such as *Oct4*, *Nanog*, and *Tbx3*, demonstrating that depletion of SMYD5 leads to compromised self-renewal. However, modulation of OCT4 levels did not diminish the impact of depleting SMYD5 in ES cells and during differentiation.

We also observed perturbed differentiation of SMYD5-depleted ES cells, where a loss of SMYD5 resulted in abnormal EB differentiation including the formation of complex structures containing circular bulges lined with a PE, and expression of endodermal genes such as *Sox17*. The differential formation of endoderm between control and shSmyd5 cells was also visible in teratomas. Moreover, our results describing an important role for the H4K20 histone methyltransferase, SMYD5, in ES cell differentiation is in alignment with a previous study which demonstrated that depletion of Suv420h1/h2 histone methyltransferases leads to compromised differentiation of ES cells [49]. Combined, these findings suggest that H4K20 HMTases are important for ES cells differentiation.

### SMYD5 regulates H4K20me3 at repetitive DNA elements in ES cells

Our results support a role for SMYD5 in regulating H4K20me3 in ES cells. These results are in alignment with a previous study which implicated a role for SMYD5 as a methyltransferase that deposits H4K20me3 marks in *Drosophila* and in macrophages [19]. We found that SMYD5 binds H4K20me3-enriched regions and depletion of SMYD5 results in global decreases in H4K20me3 as evaluated by western blotting and ChIP-Seq. These results argue for a critical role of SMYD5 in regulating H4K20me3. Interestingly, our data indicated that depletion of SMYD5 also decreased levels of H3K9me3/2, G9a, and HP1α. Because H4K20me3 is known to co-localize with H3K9 methylation at heterochromatic regions [7, 50] and H3K9me3 is important for recruitment of HP1 and heterochromatin formation [51–54], it is plausible that a loss of SMYD5 and H4K20me3 may lead to decreased heterochromatin through delocalization of H3K9me3 and HP1. Along this line, HP1 isoforms have been shown to recruit Suv420h1/2, which also induce H4K20 methylation [17], suggesting that interplay between H4K20 methyltransferases, histone modifications, and HP1 proteins regulates heterochromatin. Our results showing that SMYD5 interacts with HP1α and G9a, and depletion of SMYD5 leads to decreased HP1α and G9a binding, and H3K9me3/2 levels, is in alignment with this model. Decreased H3K9me3/2 levels may be due to a disrupted interaction between SMYD5 with G9a in SMYD5-depleted ES cells, as G9a deposits H3K9 methylation marks and is involved in regulating H3K9me3 levels in vivo [55]. It is also possible that H4K20me3 may interact with Suv39h1 or Suv39h2 histone methyltransferases, which deposit H3K9 methylation. In this case, a disrupted interaction between H4K20me3 and Suv39 h enzymes may lead to decreased H3K9 methylation levels. While we observed decreased H3K9me3 at a subset of regions (40%) in SMYD5-depleted ES cells, the majority of H3K9me3 marked-regions (60%) were unaltered (Fig. 6d), suggesting that H3K9 methylation levels change at a subset of regions in SMYD5-depleted ES cells. We also observed decreased G9a levels at a subset of islands (14%) in SMYD5-depleted ES cells (Fig. 6m), and occupancy of G9a at a subset (40%) of SMYD5 islands.

Disruption of repressive chromatin constituents of heterochromatin may trigger localized decondensation of chromatin, thus leading to de-repressed transcription of the underlying DNA. Consistent with this possibility, we observed that a decrease in H4K20me3 at LINE and LTR repetitive DNA regions by depletion of SMYD5 was accompanied by decreased levels of the heterochromatin mark H3K9me3 and increased expression of LINE and LTR repetitive DNA elements. Moreover, in addition to observing a redistribution of H3K9me3 levels in shSmyd5 ES cells using ChIP-Seq, we performed H3K9me3 immunofluorescence analysis using shLuc and shSmyd5 ES cells (see Additional file 10: Figure S10A, B) and observed decreased H3K9me3 heterochromatin foci in shSmyd5 ES cells (see Additional file 10: Figure S10C), further suggesting that depletion of SMYD5 leads to a relaxed chromatin state. While we observed a co-occurrence of the repressive histone modifications H4K20me3 and H3K9me3 at LINE and LTR repetitive elements, the role for multiple heterochromatin-associated histone modifications at repetitive genomic regions is not fully known. A possible explanation for the co-occurrence is that H4K20me3 and H3K9me3 may serve as redundant markers to facilitate chromatin compaction and maintenance of heterochromatin. Another explanation is that H4K20me3 and H3K9me3 may interact with a broader set of repressors compared with H4K20me3 or H3K9me3 alone. As such, combinatorial marking by H4K20me3 and H3K9me3 may provide greater repressive abilities relative to H4K20me3 or H3K9me3. Moreover, H4K20me3 and H3K9me3 may facilitate interactions between histone modifying enzymes and heterochromatin constituents. Along this line, the H3K9 methyltransferase ESET/Setdb1 has been shown to interact with multiple repressors, including KAP1 and HP1, KAP1 has been shown to interact with ESET/Setdb1 and HP1 [56], G9a has been shown to interact with HP1, and our results demonstrate that the H4K20me3 methyltransferase SMYD5 interacts with G9a.

## Conclusions

Results presented here describe a role for SMYD5 in regulating ES cell maintenance by silencing differentiation genes. Our model suggests that repetitive DNA elements recruit SMYD5 to the vicinity of differentiation genes, thus keeping them silenced. Depletion of SMYD5 relieves the silencing of these genes and thus induces differentiation.

## Methods

### ES cell culture

R1 ES cells were cultured as previously described with minor modifications [20, 41]. Briefly, R1 ES cells were cultured on irradiated MEFs in DMEM, 15% FBS media containing LIF (ESGRO) at 37 °C with 5% CO_2_. For ChIP experiments, ES cells were cultured on gelatin-coated dishes in ES cell media containing 1.5 µM CHIR9901 (GSK3 inhibitor) for several passages to remove feeder cells. ES cells were passed by washing with PBS using serological pipets (sc-200278, sc-200280) and dissociating with trypsin. For self-renewal experiments in the absence of LIF, ES cells were cultured on gelatin-coated dishes in ES cell media without LIF and without feeders. For embryoid body (EB) formation, ES cells were cultured in low-attachment binding dishes to promote 3D formation in ES cell media without LIF. Alkaline phosphatase staining was performed using a kit from Millipore according to the manufacturer’s instructions.

### Establishment of SMYD5 expressing ES cells

R1 ES cells were nucleofected with the pEF1α-BirAV5-neo plasmid and stably selected in the presence of 300 μg/mL G418 for at least 5–7 days. Individual ES cell colonies were picked and screened for BirA expression using western blotting. An ES cell clone expressing high levels of BirA was used for the subsequent experiments. Next, Smyd5 cDNA was amplified from ES cell cDNA and cloned into the pEF1α-FLBIO-puro vector using the BamHI and XbaI sites. BirA ES cells were nucleofected with the pEF1α-FLBIO-Smyd5-puro plasmid and stably selected in the presence of 1 μg/mL puromycin and 200 μg/mL G418. Individual ES cells clones were picked and screened for SMYD5 expression using an anti-FLAG antibody and western blotting. BirA ES cells were used as a negative control for immunoprecipitation and western blotting experiments. For generation of ES cells overexpressing wild-type or mutant (H315L and C317A) SMYD5, SMYD5 was PCR-amplified from ES cell cDNA and cloned into the pCDH-neo lentiviral vector (System Biosciences).

### Lentiviral infection

ES cells were transduced with lentiviral particles encoding shRNAs as described previously [20, 41]. Briefly, shRNA template DNA oligos were annealed and double-stranded shRNA templates were cloned into the BamH1/EcoRI digested pGreenPuro Vector (System Biosciences) according to the manufacture’s protocol. To generate lentiviral particles, 293T cells were co-transfected with an envelope plasmid (pLP/VSVG), packaging vector (psPAX2), and an shRNA (shLuc or shSmyd5) or cDNA expression vector (SMYD5) using lipofectamine 2000. Twenty-four to 48 h posttransfection, the medium containing lentiviral particles was harvested, filtered, and used to infect ES cells. Twenty-four hours post-transduction, ES cells were stably selected in the presence of 1 µg/mL puromycin.

### Teratoma and tumor formation

Teratoma formation was performed as previously described [20]. Briefly, ES cells were cultured on gelatin-coated dishes to remove feeder cells, dissociated into single cells, and 10^6^ ES cells were injected subcutaneously into immunocompromised SCID–beige mice. After three to four weeks, mice were euthanized and teratomas were washed and fixed in 10% buffered formalin. Teratomas were then embedded in paraffin. Thin sections were cut and stained with hematoxylin and eosin (H&E) using standard techniques. All animals were treated in accordance with Institution Animal Care and Use Committee guidelines under current approved protocols at NHLBI.

### Q-RT-PCR expression analysis

RNA isolation and Q-RT-PCR were performed as previously described with minor modifications [20]. Briefly, RNA isolation and Q-RT-PCR were performed as previously described with minor modifications. Total RNA was harvested from ES cells using an RNeasy Mini Kit or miRNeasy Mini Kit (Qiagen, Valencia, CA) and DNase treated using Turbo DNA-free (Ambion). Reverse transcription was performed using a Superscript III kit (Invitrogen, Carlsbad, CA). Q-RT-PCR was performed using TaqMan probes, or custom FAM-labeled probes, and primers and TaqMan Universal PCR Master Mix reagents (Applied Biosystems). Primers used for Q-RT-PCR with Roche Universal probes were designed using the Universal Probe Library Assay design Center (Roche).

### Immunoflourescence analysis

ES cells were fixed with 4% paraformaldehyde for 15 min at room temperature, washed with 0.1% Triton X-100 (Sigma), and blocked in 1% BSA/0.01% Tween-20 for 30 min. Fixed cells were incubated with an anti-H3K9me3 antibody (ab8898) overnight at 4 °C in blocking buffer. The next day, the cells were washed with blocking buffer, and incubated with DAPI in 0.1% Triton X-100, washed with blocking buffer, and mounted in ProLong Gold antifade reagent (Invitrogen).

### ChIP-Seq

ChIP-Seq experiments were performed as previously described with minor modifications [20, 41, 57]. The H4K20me3 antibody (07-463) and the HP1α antibody were obtained from Millipore. The polyclonal H4K20me2 (ab9052), H4K20me1 (ab9051), H3K9me3 (ab8898), and H3K9me2 (ab1220) antibodies were obtained from Abcam. For SMYD5-FLAG ChIP-Seq, the monoclonal anti-FLAG antibody was obtained from Sigma. For SMYD5-bioChIP-Seq, streptavidin (SA) beads were obtained from Invitrogen.

Briefly, ES cells were harvested and chemically cross-linked with 1% formaldehyde (Sigma) for 5–10 min at 37 °C and subsequently sonicated. Sonicated cell extracts were used for ChIP assays. ChIP-enriched DNA was end-repaired using the End-It DNA End-Repair kit (Epicentre), followed by addition of a single A nucleotide, and ligation of PE adapters (Illumina) or custom-indexed adapters. PCR was performed using Phusion High-Fidelity PCR master mix. ChIP libraries were sequenced on an Illumina HiSeq platform according to the manufacture’s protocol.

Sequence reads were mapped to the mouse genome (mm9) using Bowtie2 [58]. To allow mapping to repetitive elements, we used the default mode of Bowtie2, which searches for multiple alignments, and reports the best one based on the alignment score (MAPQ) (http://bowtie-bio.sourceforge.net/bowtie2/manual.shtml).

ChIP-Seq read-enriched regions were identified relative to Input DNA (sonicated chromatin) as previously described with minor modifications [59, 60]. Briefly, ChIP-Seq read-enriched regions (peaks) were identified relative to Input DNA using “Spatial Clustering for Identification of ChIP-Enriched Regions” (SICER) software [60] with a window size setting of 200 bps, a gap setting of 400 bps, and a FDR setting of 0.001. For a comparison of ChIP-enrichment between samples, a fold-change threshold of 1.5 and an FDR setting of 0.001 were used. For transcription factors (see Fig. 3g), the ChIP-Seq read-enriched peaks were called by MACS [61] with a *p* value setting of 0.00001. The RPBM measure (read per base per million reads) was used to quantify the density of histone modification, SMYD5 binding, and Input DNA at genomic regions from ChIP-Seq datasets. We have also applied the Kolmogorov–Smirnov test to obtain *p* value statistics and compare densities at genomic regions.

### reChIP

reChIP, also termed sequential ChIP, was performed as previously described with minor modifications [32]. Cross-linked chromatin from ES cells was immunoprecipitated with antibodies against either H4K20me3 or FLAG (for SMYD5) as described above (see “ChIP-Seq”), except that chromatin was eluted in a TE solution containing 20 mM DTT, 500 mM NaCL, and 1% SDS at 37° for 20 min. The eluted DNA was diluted 50-fold, and a second round of immunoprecipitations was performed against the FLAG or H4K20me3 antibody as described above. PCR primers for evaluating reChIP were designed from the indicated genomic regions. Real-time PCR was performed using an Applied Biosystems OneStepPlus machine. For reChIP, 1 µL of reChIP DNA or 1 µL of Input DNA was used as a template, and relative enrichment was determined from a standard curve for each primer using a standard curve of Input DNA, and using the Nanog promoter (which does not contain enrichment of H4K20me3 or SMYD5) as a normalizer.

### RNA-Seq analysis

RNA was harvested from ES cells and EBs as described above. RNA-Seq was performed as previously described [20, 41]. RNA was harvested from ES cells and EBs as described above. mRNA was purified using a Dynabeads mRNA purification kit (Invitrogen). Double-stranded cDNA was generated using a SuperScript Double-Stranded cDNA synthesis kit (Invitrogen). cDNA was end-repaired using the End-It DNA End-Repair kit (Epicentre), followed by addition of a single A nucleotide, and ligation of PE adapters (Illumina) or custom-indexed adapters. PCR was performed using Phusion High-Fidelity PCR master mix. RNA-Seq libraries were sequenced on Illumina GAIIX or HiSeq platforms according to the manufacture’s protocol.

The RPKM measure (reads per kilo bases of exon model per million reads) proposed previously [62] was used to quantify the mRNA expression level of a gene from RNA-Seq datasets. Differentially expressed genes were identified using EdgeR (FDR < 0.001 and FC > 2) [63]. Genes with RPKM < 3 in both conditions in comparison were excluded from this analysis.

### Prediction of differentially expressed genes due to chance or accelerated differentiation

Expression changes that indicate accelerated differentiation include genes that show accelerated downregulation or upregulation during EB differentiation. The percentage of genes that lag behind during differentiation of shSmyd5 ES cells is less than expected. Each bar represents a group of genes upregulated by at least alpha-fold (*X* axis) from ESC (0 h) to EB day 6 in the control cells. The percentage of genes with expression values that follow the order: EB day 6 (shLuc) > EB day 6 (shSmyd5) > ES cell is calculated (Observed; red bars); error bars are generated by bootstrapping. The expression values of all genes are randomly shuffled independently for EB day 6 (shLuc), EB day 6 (shSmyd5), and ES cells and are repeated many times to give the means and standard deviations for the expectations (Expected; blue bars). The red bars represent observed data.

### Annotation of repetitive DNA sequences

Repetitive DNA sequence classes (e.g., LINE, LTR), families (L1, ERVK), and names (e.g., L1Md_T, IAPLTR1) for the mm9 reference genome were defined according to the annotations provided by the UCSC genome browser and RepeatMasker (http://www.repeatmasker.org), which uses curated libraries of repeats such as Repbase (http://www.girinst.org/repbase/).

### In vitro pull-down assay

Nuclear extracts were prepared from ES cells using a standard high salt extraction protocol [64]. Briefly, cells were lysed by Dounce homogenizing in buffer A, washed, and nuclear proteins were extracted with buffer C. The salt concentration was diluted as described [65], and incubated with histone peptides (unmodified or modified) prebound to avidin beads overnight at 4 °C. Beads were washed, eluted, and analyzed by SDS-PAGE.

## Additional files

**Additional file 1: Figure S1.** Depletion of SMYD5 leads to decreased self-renewal and altered differentiation. **(A)** RNA-Seq data of Smyd5 expression in ES cells and day 14 differentiated embryoid bodies (EBs; log2 RPKM). **(B)** Q-RT-PCR analysis of *Smyd5* expression in control (shLuc) ES cells and SMYD5 shRNA knockdown ES cells (shSmyd5-1, shSmyd5-2, and shSmyd5-3). **(C)** Bright-field microscopy of ES cells infected with shLuc (control) or shSmyd5 lentiviral particles (shSmyd5-1, shSmyd5-2, and shSmyd5-3) and stably selected with puromycin. **(D)** Q-RT-PCR expression analysis during shLuc and shSmyd5 EB differentiation.

**Additional file 2: Figure S2.** SMYD5 and H4K20me3 co-occupancy in ES cells. (**A**) Venn diagram showing comparison of SMYD5-FLAG and SMYD5-bioChIP ChIP-enriched peaks. (**B**) Heat map of SMYD5-FLAG and FLAG-bioChIP ChIP-Seq densities at FLAG-bioChIP intersecting regions. (**C**) Average profiles of SMYD5-bioChIP and SMYD5-FLAG density at FLAG-bioChIP intersecting enriched regions. (**D**) Western blot of H4K20me3 in shLuc and shSmyd5 ES cells.

**Additional file 3: Figure S3.** SMYD5 and H4K20me3 are enriched at LINE and LTR repeats in ES cells. **(A)** Repetitive DNA sequences (LINE and LTR) are enriched in H4K20me3, H3K9me3 and SMYD5 genomic sites. Comparison of H4K20me3, H3K9me3, and SMYD5 enriched sequences and annotated repetitive sequences (http://www.repeatmasker.org). The percentage of ChIP-enriched regions with at least 60% repeat length is shown. Note the predominance of LTR and LINE repetitive DNA sequences in ChIP-enriched islands. **(B)** Repeat subfamilies belonging to the LINE and LTR repetitive DNA sequence classes are enriched in H4K20me3, H3K9me3 and SMYD5 genomic sites. The percentage of ChIP-enriched regions with at least 60% repeat length is shown. **(C)** Repetitive DNA sequence family members L1 and ERVK are enriched in H4K20me3, H3K9me3 and SMYD5 genomic regions.

**Additional file 4: Figure S4.** Enrichment of LINE and LTR repeat subfamilies at SMYD5, H4K20me3, and H3K9me3 occupied regions. **(A, C)** Hierarchical clustering heat map showing the percent coverage of repeat subfamilies belonging to the LINE and LTR class within (**A**) H4K20me3 (**B**) H3K9me3, and (**C**) SMYD5 ChIP-enriched regions. Red indicates an elevated percent coverage of a repeat element. The *X* axis shows the H4K20me3, H3K9me3, and SMYD5 ChIP-peaks while the *Y* axis shows the LINE or LTR element name.

**Additional file 5: Figure S5.** H4K20me3 density at SMYD5 bound regions. **(A)** Empirical cumulative distribution for the density of H4K20me3 at SMYD5-enriched regions in shLuc and shSmyd5 ES cells. The boxplot shows the density of H4K20me3 at SMYD5-enriched regions (log2 fold-change vs. Input) in shLuc and shSmyd5 ES cells.

**Additional file 6: Figure S6.** Re-ChIP Validation of H4K20me3 and SMYD5 co-occupancy in ES cells. **(A)** Real-time PCR depicting the relative enrichment of H4K20me3/SMYD5 co-occupied or control (Nanog promoter) genomic sites after sequential immunoprecipitations with an anti-FLAG antibody (for SMYD5) and then an anti-H4K20me3 antibody, or an anti-H4K20me3 antibody and then an anti-FLAG antibody. (**B**) Fold-change enrichment of H4K20me3/SMYD5 co-occupied genomic sites relative to the control (Nanog promoter) site. Region #10 is also depicted in Fig. 7g. (**C**) Annotation of H4K20me3/SMYD5 regions using HOMER software [69].

**Additional file 7: Figure S7.** Depletion of SMYD5 leads to decreased H4K20me3, H3K9me3, and HP1α at LINE/LTR repeats. **(A)** Peptide pull-down assays using ES cell nuclear extracts and unmodified or modified H4/H3 peptides were performed and analyzed by immunoblotting with anti-HP1α, anti-G9a, and anti-ESET antibodies. **(B)** Empirical cumulative distribution for the density of H4K20me3 (top left panel), H3K9me3 (bottom left panel), HP1α (bottom right panel) at LINE regions in shLuc and shSmyd5 ES cells, and SMYD5-FLAG in ES cells (top right panel). **(C)** Empirical cumulative distribution for the density of H4K20me3 (top left panel), H3K9me3 (bottom left panel), HP1α (bottom right panel) at LTR regions in shLuc and shSmyd5 ES cells, and SMYD5-FLAG in ES cells (top right panel).

**Additional file 8: Figure S8.** Elevated expression of repetitive DNA elements in SMYD5 knockdown ES cells. Browser view of RNA-Seq expression and H4K20me3, and H3K9me3 in shLuc and shSmyd5 ES cells, and SMYD5-FLAG and SMYD5-bioChIP in ES cells. The green box highlights a genomic region enriched with H4K20me3 and other histone modifications in control (shLuc) ES cells.

**Additional file 9: Figure S9.** Upregulated genes in SMYD5 knockdown cells associated with LTR/ERV elements and decreased H4K20me3. Loss of SMYD5-dependent silencing of LTR/ERV elements influences the expression of nearby genes. **(A)** Number of differentially expressed (DE) genes between shLuc and shSmyd5 ES cells (fold-change >1.5, *p* value <0.05). **(B)** Expression of upregulated genes between shLuc and shSmyd5 ES cells (*p* = 5.528e−13) (log2 RPKM). **(C)** Annotation of LTR/ERV elements nearby DE genes in shLuc and shSmyd5 ES using HOMER software [69]. **(D)** Fold-change expression of LTR/ERV elements at DE genes (A-C) relative to total mRNA in shSmyd5 ES cells relative to shLuc ES cells. **(E)** Density of H4K20me3 marks nearby LTR/ERV element and within 10 kb of TSS of DE genes. **(F)** Mouse gene atlas expression analysis evaluated using Network2Canvas [66] demonstrates that lineage and ES cell genes are misexpressed in shSmyd5 ES cells. Each node (square) represents a gene list (shLuc vs shSmyd5 DE genes bound by SMYD5 and containing LTR/LINE element) associated with a gene-set library (mouse gene atlas). The brightness (white) of each node is determined by its *p* value.

**Additional file 10: Figure S10.** Decreased H3K9me3 heterochromatin foci in SMYD5-depleted ES cells. **(A, B)** Immunofluorescence staining of H3K9me3 in (**A**) shLuc and (**B**) shSmyd5 ES cells. Nuclei were stained with DAPI. (**C**) Quantitation of nuclear H3K9me3 heterochromatin foci using ImageJ software.

## Abbreviations

ES cells: embryonic stem cells
H4K20me3: trimethylated histone 4 lysine 20
H3K9me3: trimethylated histone 3 lysine 9
SMYD5: set and mynd domain 5
LTR: long terminal repeat
LINE: long interspersed nuclear element
shRNA: short hairpin RNA
ERV: endogenous retrovirus
TF: transcription factor
HP1: heterochromatin protein 1
RNA-Seq: RNA sequencing
WT: wild-type
3D: three dimensional
AP: alkaline phosphatase
DE: differentially expressed
Q-RT-PCR: quantitative real-time PCR
GSEA: gene set enrichment analysis
GO: gene ontology
LIF: leukemia inhibitory factor
EB: embryoid body
PE: primitive endoderm
ICM: inner cell mass
H&E: hematoxylin and eosin
PCA: principle component analysis
bioChIP: biotin-mediated ChIP
SICER: spatial clustering for identification of ChIP-enriched regions
ChIP-Seq: chromatin immunoprecipitation sequencing
reChIP: sequential ChIP
kb: kilobase
TSS: transcription start site
SCID: sever combined immunodeficiency
FDR: false discovery rate
MACS: model-based analysis for ChIP-Seq
RPKM: reads per kilo bases of exon model per million reads
RPBM: read per base per million reads
