# Supplementary figures and images for: SMYD5 regulates H4K20me3-marked heterochromatin to safeguard ES cell self-renewal and prevent spurious differentiation
Source: Epigenetics Chromatin. 2017 Feb 23;10:8. doi: 10.1186/s13072-017-0115-7 (PMC5324308; doi:10.1186/s13072-017-0115-7)

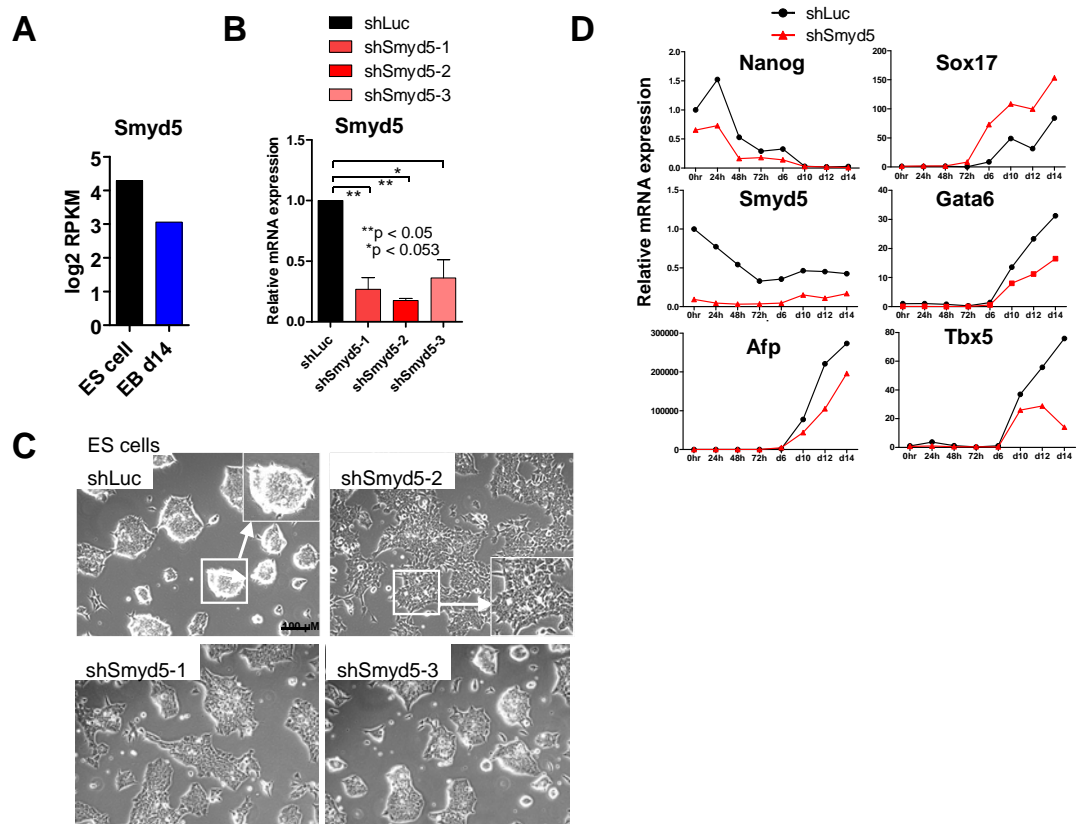

**Figure S1**

Supplement: Supplementary file 1 — Additional file 1: Figure S1. Depletion of SMYD5 leads to decreased self-renewal and altered differentiation. (A) RNA-Seq data of Smyd5 expression in ES cells and day 14 differentiated embryoid bodies (EBs; log2 RPKM). (B) Q-RT-PCR analysis of Smyd5 expression in control (shLuc) ES cells and SMYD5 shRNA knockdown ES cells (shSmyd5-1, shSmyd5-2, and shSmyd5-3). (C) Bright-field microscopy of ES cells infected with shLuc (control) or shSmyd5 lentiviral particles (shSmyd5-1, shSmyd5-2, and shSmyd5-3) and stably selected with puromycin. (D) Q-RT-PCR expression analysis during shLuc and shSmyd5 EB differentiation. [file 13072_2017_115_MOESM1_ESM.pdf]

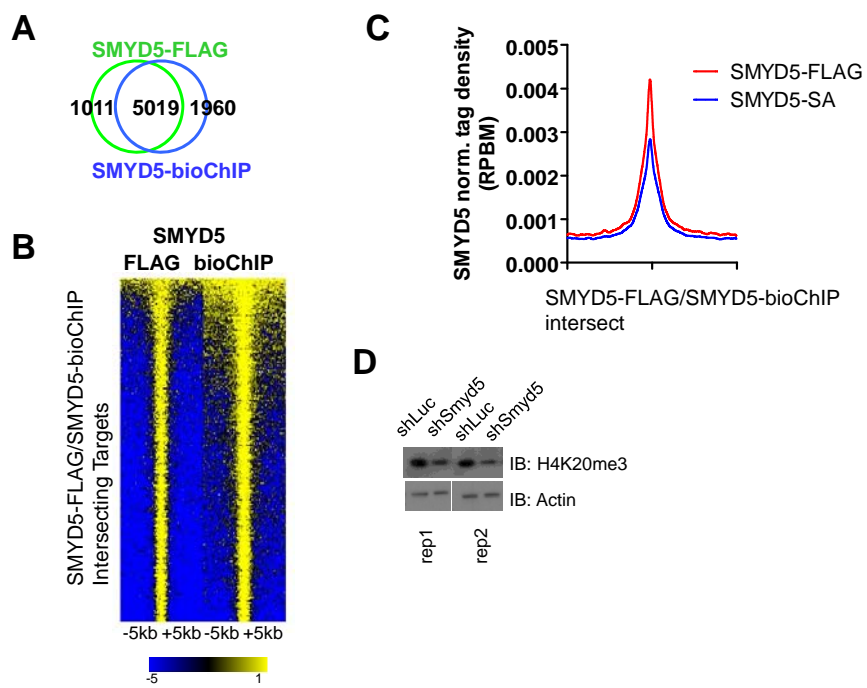

**Figure S2**

Supplement: Supplementary file 2 — Additional file 2: Figure S2. SMYD5 and H4K20me3 co-occupancy in ES cells. (A) Venn diagram showing comparison of SMYD5-FLAG and SMYD5-bioChIP ChIP-enriched peaks. (B) Heat map of SMYD5-FLAG and FLAG-bioChIP ChIP-Seq densities at FLAG-bioChIP intersecting regions. (C) Average profiles of SMYD5-bioChIP and SMYD5-FLAG density at FLAG-bioChIP intersecting enriched regions. (D) Western blot of H4K20me3 in shLuc and shSmyd5 ES cells. [file 13072_2017_115_MOESM2_ESM.pdf]

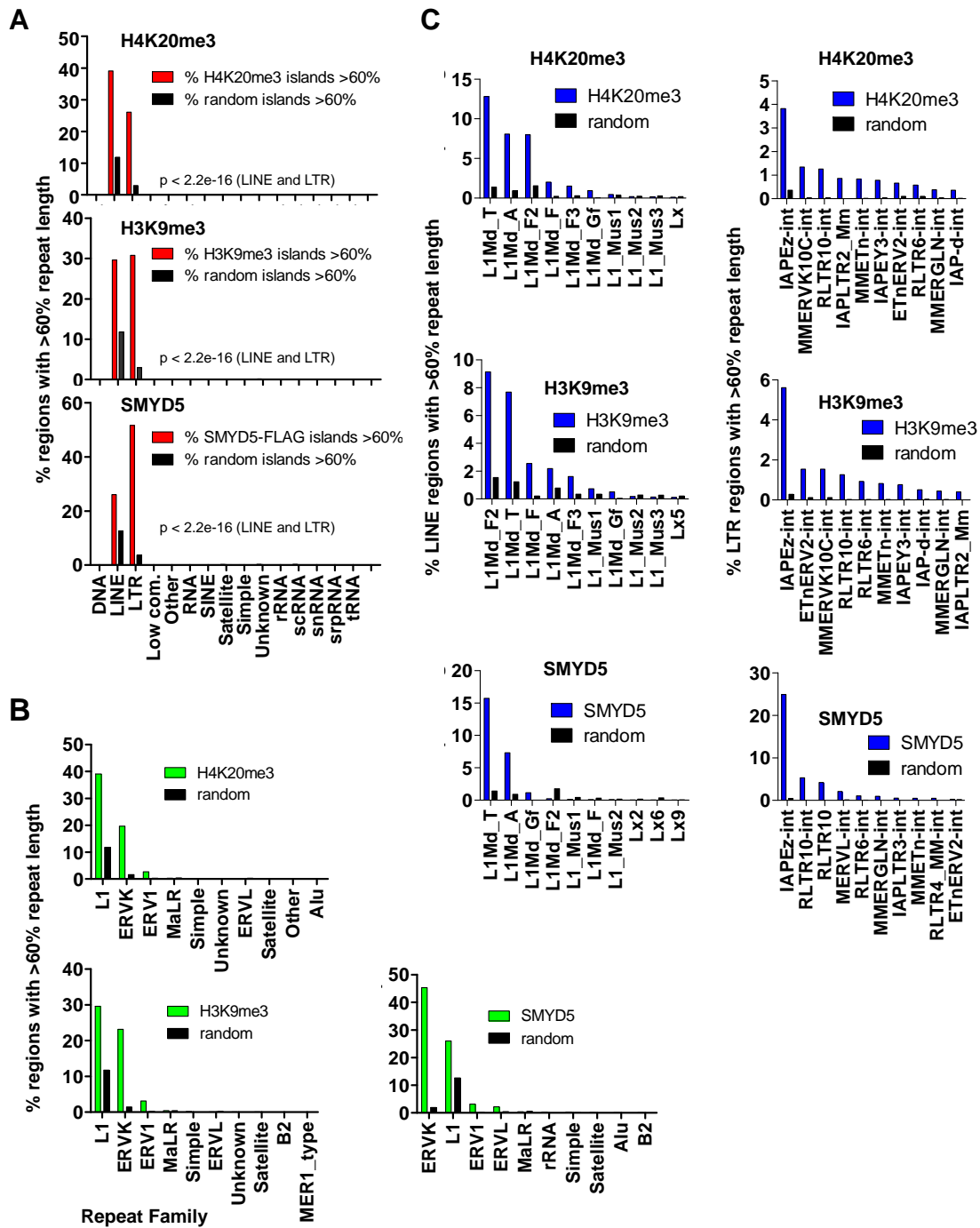

Figure S3

Supplement: Supplementary file 3 — Additional file 3: Figure S3. SMYD5 and H4K20me3 are enriched at LINE and LTR repeats in ES cells. (A) Repetitive DNA sequences (LINE and LTR) are enriched in H4K20me3, H3K9me3 and SMYD5 genomic sites. Comparison of H4K20me3, H3K9me3, and SMYD5 enriched sequences and annotated repetitive sequences (http://www.repeatmasker.org). The percentage of ChIP-enriched regions with at least 60% repeat length is shown. Note the predominance of LTR and LINE repetitive DNA sequences in ChIP-enriched islands. (B) Repeat subfamilies belonging to the LINE and LTR repetitive DNA sequence classes are enriched in H4K20me3, H3K9me3 and SMYD5 genomic sites. The percentage of ChIP-enriched regions with at least 60% repeat length is shown. (C) Repetitive DNA sequence family members L1 and ERVK are enriched in H4K20me3, H3K9me3 and SMYD5 genomic regions. [file 13072_2017_115_MOESM3_ESM.pdf]

**A****H4K20me3 SICER Islands**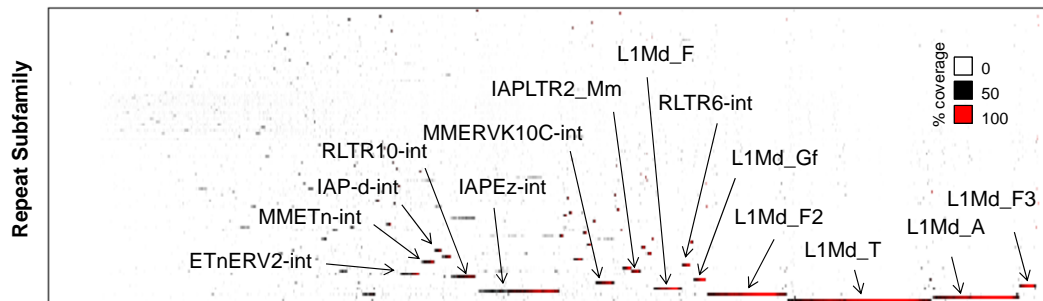**B****H3K9me3 SICER Islands**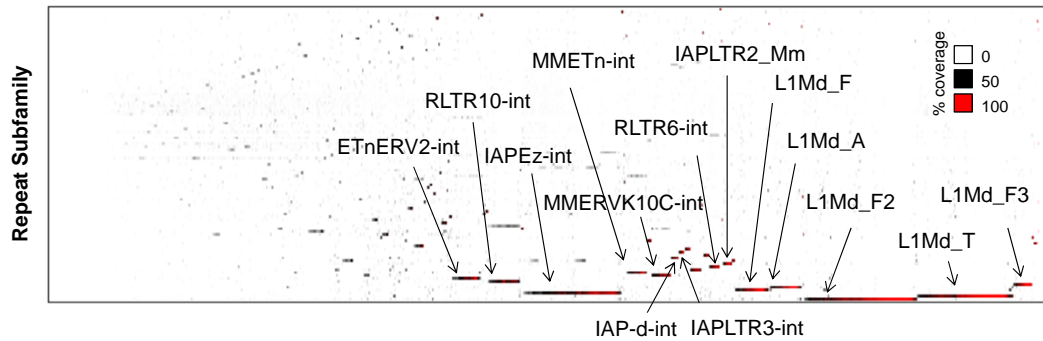**C****SMYD5 SICER Islands**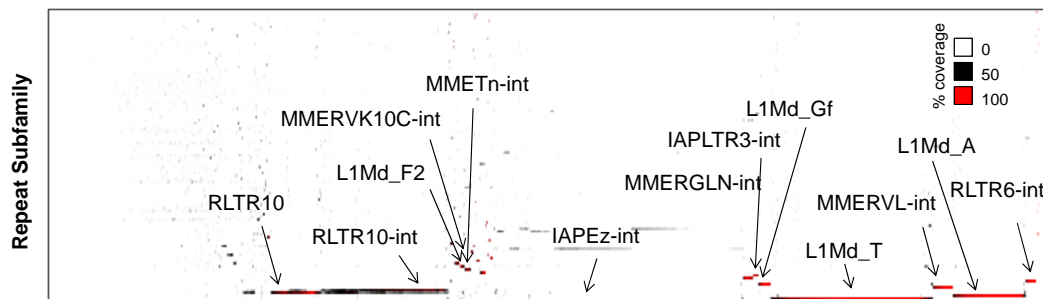**Figure S4**

Supplement: Supplementary file 4 — Additional file 4: Figure S4. Enrichment of LINE and LTR repeat subfamilies at SMYD5, H4K20me3, and H3K9me3 occupied regions. (A, C) Hierarchical clustering heat map showing the percent coverage of repeat subfamilies belonging to the LINE and LTR class within (A) H4K20me3 (B) H3K9me3, and (C) SMYD5 ChIP-enriched regions. Red indicates an elevated percent coverage of a repeat element. The X axis shows the H4K20me3, H3K9me3, and SMYD5 ChIP-peaks while the Y axis shows the LINE or LTR element name. [file 13072_2017_115_MOESM4_ESM.pdf]

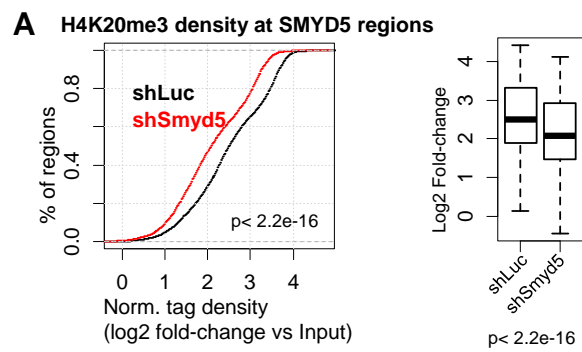

**Figure S5**

Supplement: Supplementary file 5 — Additional file 5: Figure S5. H4K20me3 density at SMYD5 bound regions. (A) Empirical cumulative distribution for the density of H4K20me3 at SMYD5-enriched regions in shLuc and shSmyd5 ES cells. The boxplot shows the density of H4K20me3 at SMYD5-enriched regions (log2 fold-change vs. Input) in shLuc and shSmyd5 ES cells. [file 13072_2017_115_MOESM5_ESM.pdf]

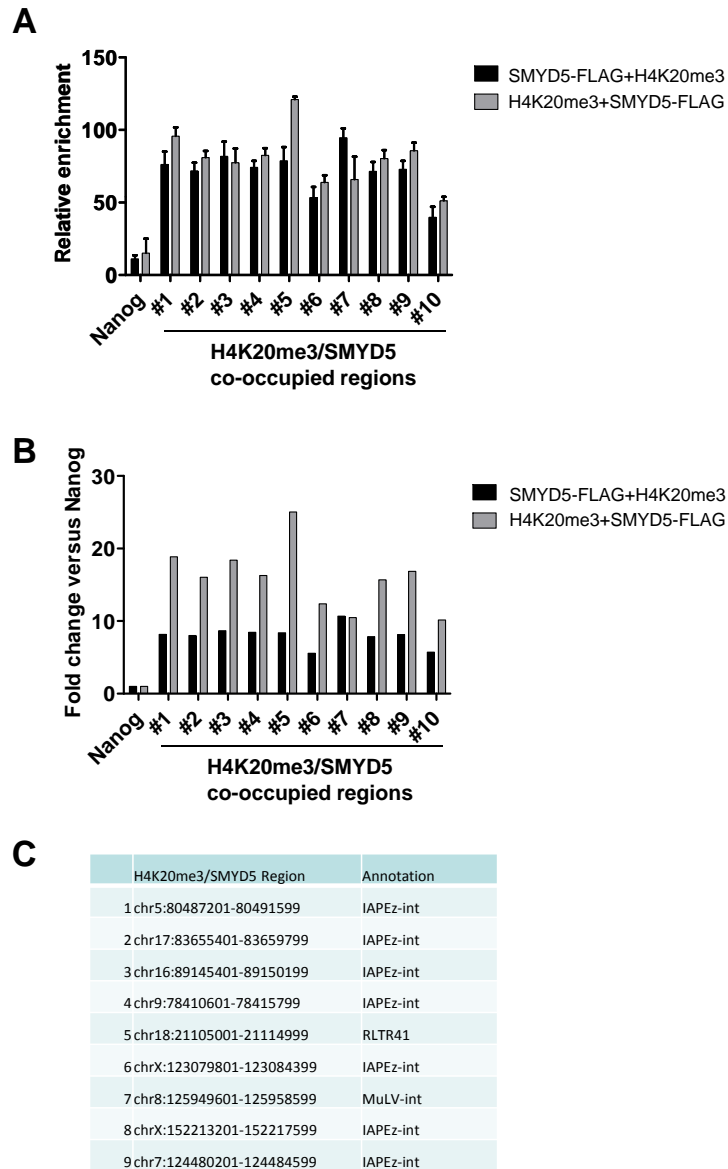

**Figure S6**

Supplement: Supplementary file 6 — Additional file 6: Figure S6. Re-ChIP Validation of H4K20me3 and SMYD5 co-occupancy in ES cells. (A) Real-time PCR depicting the relative enrichment of H4K20me3/SMYD5 co-occupied or control (Nanog promoter) genomic sites after sequential immunoprecipitations with an anti-FLAG antibody (for SMYD5) and then an anti-H4K20me3 antibody, or an anti-H4K20me3 antibody and then an anti-FLAG antibody. (B) Fold-change enrichment of H4K20me3/SMYD5 co-occupied genomic sites relative to the control (Nanog promoter) site. Region #10 is also depicted in Fig. 7g. (C) Annotation of H4K20me3/SMYD5 regions using HOMER software [69]. [file 13072_2017_115_MOESM6_ESM.pdf]

**A**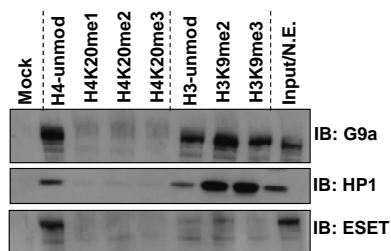**B**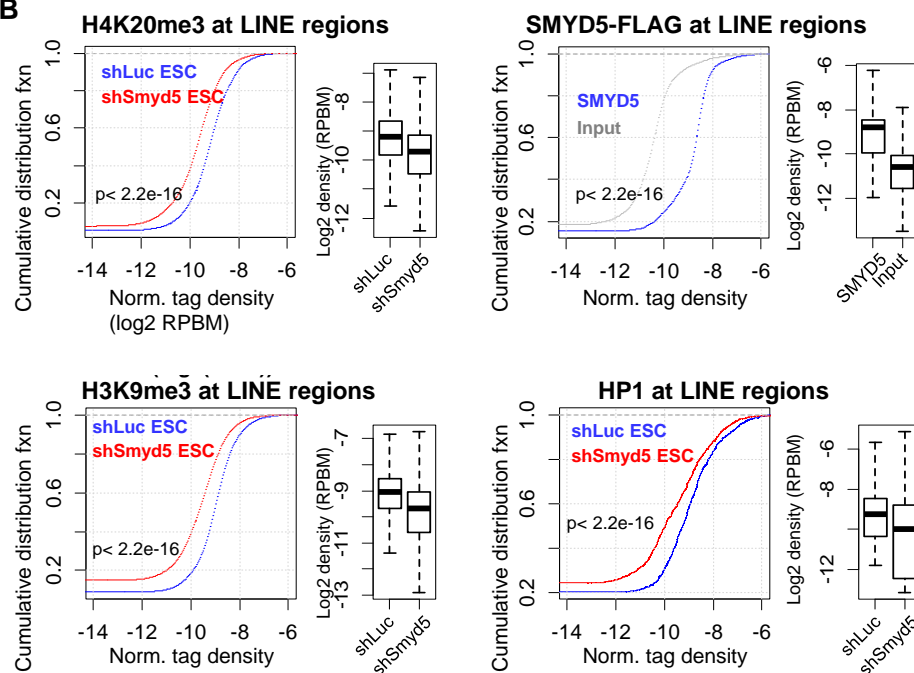**C**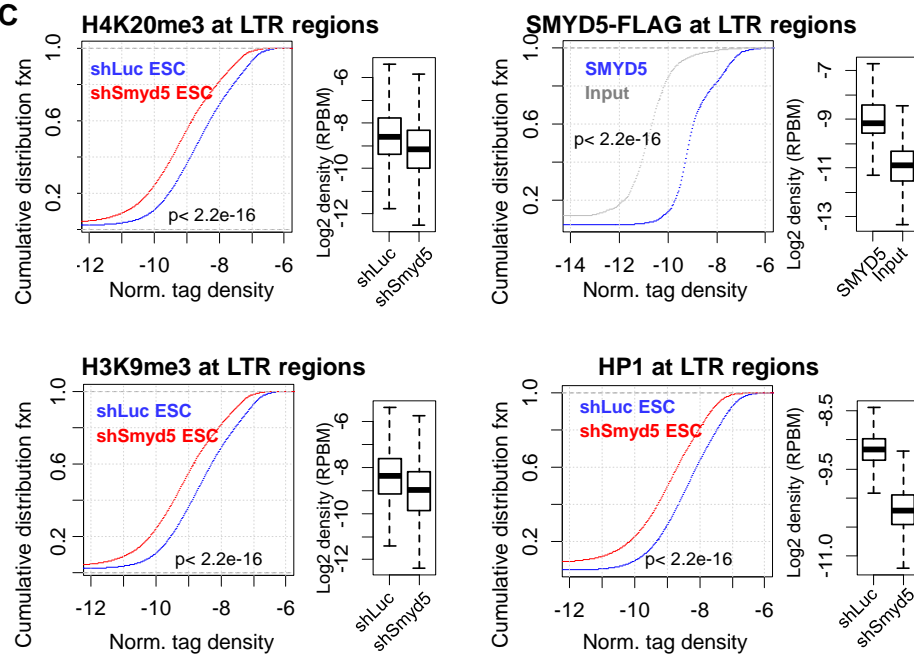**Figure S7**

Supplement: Supplementary file 7 — Additional file 7: Figure S7. Depletion of SMYD5 leads to decreased H4K20me3, H3K9me3, and HP1α at LINE/LTR repeats. (A) Peptide pull-down assays using ES cell nuclear extracts and unmodified or modified H4/H3 peptides were performed and analyzed by immunoblotting with anti-HP1α, anti-G9a, and anti-ESET antibodies. (B) Empirical cumulative distribution for the density of H4K20me3 (top left panel), H3K9me3 (bottom left panel), HP1α (bottom right panel) at LINE regions in shLuc and shSmyd5 ES cells, and SMYD5-FLAG in ES cells (top right panel). (C) Empirical cumulative distribution for the density of H4K20me3 (top left panel), H3K9me3 (bottom left panel), HP1α (bottom right panel) at LTR regions in shLuc and shSmyd5 ES cells, and SMYD5-FLAG in ES cells (top right panel). [file 13072_2017_115_MOESM7_ESM.pdf]

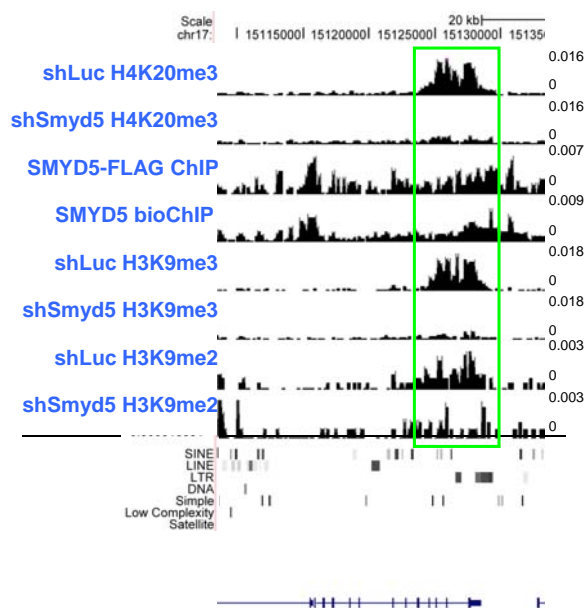

Figure S8

Supplement: Supplementary file 8 — Additional file 8: Figure S8. Elevated expression of repetitive DNA elements in SMYD5 knockdown ES cells. Browser view of RNA-Seq expression and H4K20me3, and H3K9me3 in shLuc and shSmyd5 ES cells, and SMYD5-FLAG and SMYD5-bioChIP in ES cells. The green box highlights a genomic region enriched with H4K20me3 and other histone modifications in control (shLuc) ES cells. [file 13072_2017_115_MOESM8_ESM.pdf]

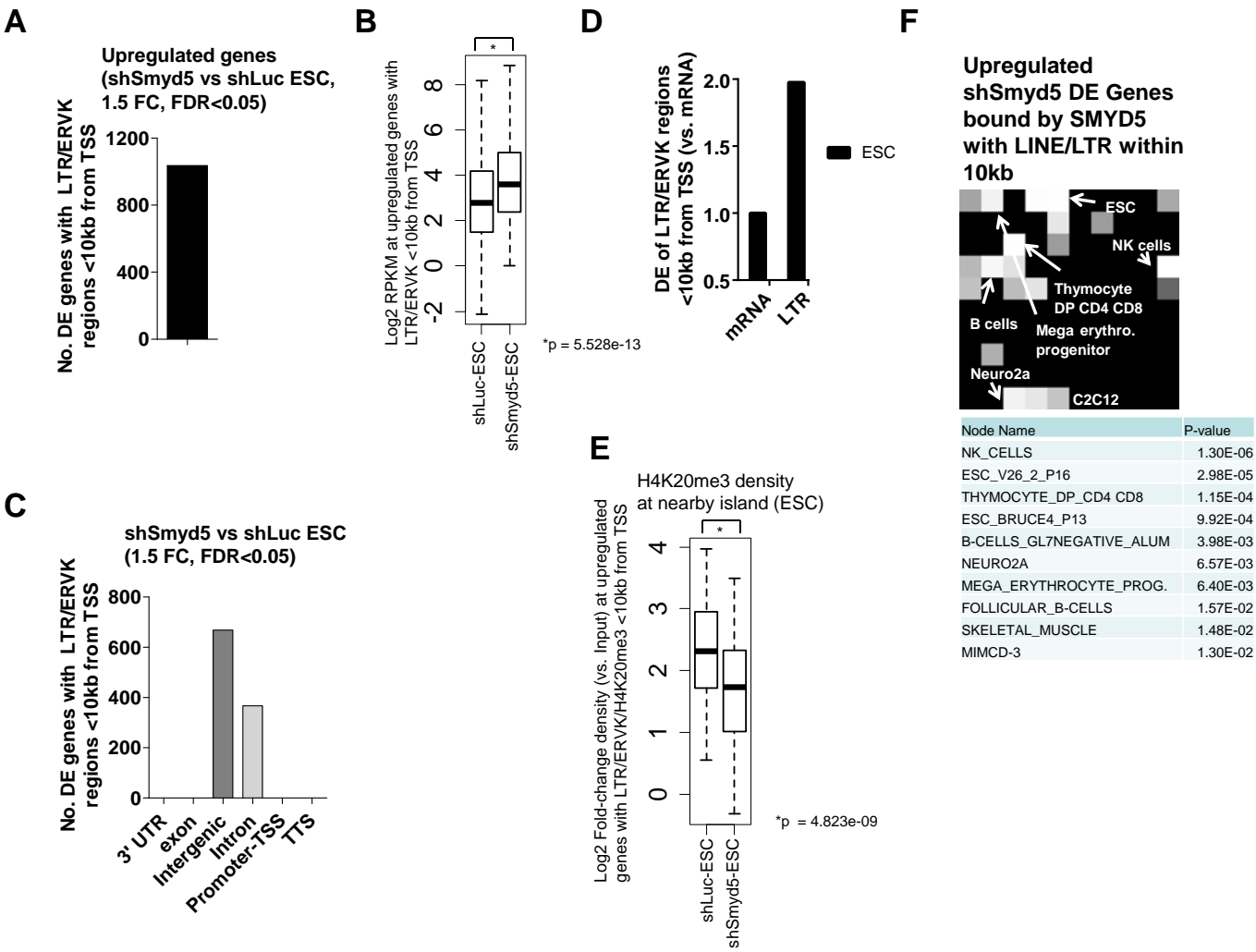

Figure S9

Supplement: Supplementary file 9 — Additional file 9: Figure S9. Upregulated genes in SMYD5 knockdown cells associated with LTR/ERV elements and decreased H4K20me3. Loss of SMYD5-dependent silencing of LTR/ERV elements influences the expression of nearby genes. (A) Number of differentially expressed (DE) genes between shLuc and shSmyd5 ES cells (fold-change >1.5, p value <0.05). (B) Expression of upregulated genes between shLuc and shSmyd5 ES cells (p = 5.528e−13) (log2 RPKM). (C) Annotation of LTR/ERV elements nearby DE genes in shLuc and shSmyd5 ES using HOMER software [69]. (D) Fold-change expression of LTR/ERV elements at DE genes (A-C) relative to total mRNA in shSmyd5 ES cells relative to shLuc ES cells. (E) Density of H4K20me3 marks nearby LTR/ERV element and within 10 kb of TSS of DE genes. (F) Mouse gene atlas expression analysis evaluated using Network2Canvas [66] demonstrates that lineage and ES cell genes are misexpressed in shSmyd5 ES cells. Each node (square) represents a gene list (shLuc vs shSmyd5 DE genes bound by SMYD5 and containing LTR/LINE element) associated with a gene-set library (mouse gene atlas). The brightness (white) of each node is determined by its p value. [file 13072_2017_115_MOESM9_ESM.pdf]

**A**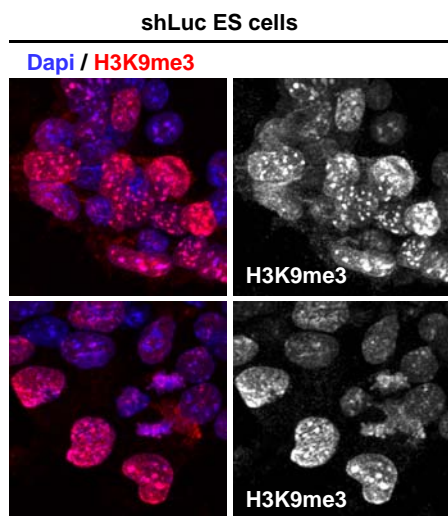**B**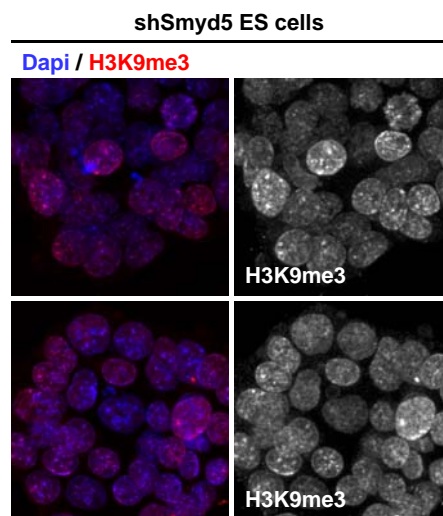**C**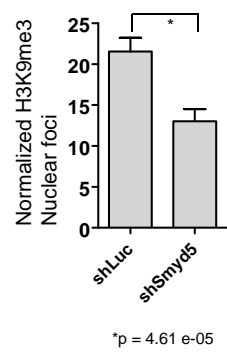**Figure S10**

Supplement: Supplementary file 10 — Additional file 10: Figure S10. Decreased H3K9me3 heterochromatin foci in SMYD5-depleted ES cells. (A, B) Immunofluorescence staining of H3K9me3 in (A) shLuc and (B) shSmyd5 ES cells. Nuclei were stained with DAPI. (C) Quantitation of nuclear H3K9me3 heterochromatin foci using ImageJ software. [file 13072_2017_115_MOESM10_ESM.pdf]
